# The Onion (*Allium cepa* L.) *R2R3-MYB* Gene *MYB1* Regulates Anthocyanin Biosynthesis

**DOI:** 10.3389/fpls.2016.01865

**Published:** 2016-12-09

**Authors:** Kathy E. Schwinn, Hanh Ngo, Fernand Kenel, David A. Brummell, Nick W. Albert, John A. McCallum, Meeghan Pither-Joyce, Ross N. Crowhurst, Colin Eady, Kevin M. Davies

**Affiliations:** ^1^The New Zealand Institute for Plant & Food Research LimitedPalmerston North, New Zealand; ^2^The New Zealand Institute for Plant & Food Research LimitedChristchurch, New Zealand; ^3^The New Zealand Institute for Plant & Food Research LimitedAuckland, New Zealand

**Keywords:** onion, garlic, R2R3-MYB, anthocyanin, regulation, Asparagales, transgenic

## Abstract

Bulb color is an important consumer trait for onion (*Allium cepa* L., Allioideae, Asparagales). The bulbs accumulate a range of flavonoid compounds, including anthocyanins (red), flavonols (pale yellow), and chalcones (bright yellow). Flavonoid regulation is poorly characterized in onion and in other plants belonging to the Asparagales, despite being a major plant order containing many important crop and ornamental species. R2R3-MYB transcription factors associated with the regulation of distinct branches of the flavonoid pathway were isolated from onion. These belonged to sub-groups (SGs) that commonly activate anthocyanin (SG6, MYB1) or flavonol (SG7, MYB29) production, or repress phenylpropanoid/flavonoid synthesis (SG4, MYB4, MYB5). *MYB1* was demonstrated to be a positive regulator of anthocyanin biosynthesis by the induction of anthocyanin production in onion tissue when transiently overexpressed and by reduction of pigmentation when transiently repressed via RNAi. Furthermore, ectopic red pigmentation was observed in garlic (*Allium sativum* L.) plants stably transformed with a construct for co-overexpression of *MYB1* and a *bHLH* partner. *MYB1* also was able to complement the acyanic petal phenotype of a defined *R2R3-MYB* anthocyanin mutant in *Antirrhinum maju*s of the asterid clade of eudicots. The availability of sequence information for flavonoid-related MYBs from onion enabled phylogenetic groupings to be determined across monocotyledonous and dicotyledonous species, including the identification of characteristic amino acid motifs. This analysis suggests that divergent evolution of the R2R3-MYB family has occurred between Poaceae/Orchidaceae and Allioideae species. The DNA sequences identified will be valuable for future analysis of classical flavonoid genetic loci in *Allium* crops and will assist the breeding of these important crop species.

## Introduction

Alliums have been used for culinary purposes for over 5000 years, and bulb onion (*Allium cepa* L.) is currently one of the most widely cultivated of all crop plants. It belongs to the Asparagales, a major plant order accounting for around 29,000 of the approximately 60,000 known monocotyledonous species, including those of the families Amaryllidaceae, Alliaceae (now the Allioideae), Asparagaceae, Hemerocallidaceae, and Orchidaceae. The genus *Allium* is in the Allioideae sub-family within in the family Amaryllidaceae, which is part of the ‘core’ Asparagales (Angiosperm Phylogeny Group III, 2009). Despite containing many economically important species, the Asparagales are poorly characterized at the molecular genetic level compared with most leading crop plants. One reason for this is the frequent occurrence of large genome sizes in these plant families (Leitch et al., 2009). Indeed, onion has one of the largest genomes among crop plants, with its 17 pg (16 Gb) haploid nuclear genome being more than 100-fold larger than that of Arabidopsis.

Color is a key breeding trait in onion, both for the visual appeal to consumers and the increasing association between plant pigments and human health benefits (Martin et al., 2011; Davies and Espley, 2013). Bulbs may be white, yellow, gold, pink, or red, because of variation in the production of different flavonoid compounds: red anthocyanins, pale yellow flavonols, and bright yellow chalcones. There is also a rare ‘chartreuse’ color containing unidentified green compounds (Green et al., 1997; Khar et al., 2008). Onion bulb color is the result of the interaction of at least five major loci – *I, C, G, L*, and *R*. Colouration requires recessive alleles for the *I* locus, which has a dominant white action, and at least one functional allele for the *C* locus (Kim et al., 2005b). The other loci have modifying effects on the yellow, red, or chartreuse colouration. The basis of the recessive *g* character, for chartreuse, is not known, although the possible involvement of chalcones has been suggested (Kim et al., 2004c). DNA sequences for several of the flavonoid biosynthetic genes have been identified in onion, and some of these have been assigned to specific chromosomes (Masuzaki et al., 2006). This has enabled some of the classical genetic loci affecting bulb color to be defined. The *L* locus has been shown to correspond to an anthocyanidin synthase (*ANS*) gene (Kim et al., 2005a). A non-functional mutant allele (*ANS-l*) accounts for the difference between yellow and red Brazilian-type onions (Kim et al., 2005a), and a second allele with reduced transcription (*ANS-p*, sometimes referred to as the *P* locus) produces pink colouration (Kim et al., 2004b). Mutations within the *R* locus, which encodes a dihydroflavonol 4-reductase (*DFR*) gene, account for the difference between some yellow and red ‘USA-type’ onions (Kim et al., 2004a). In addition, loss of chalcone isomerase (CHI) function results in production of gold bulbs, presumably because of the accumulation of chalcones (Kim et al., 2004c).

Anthocyanins, flavonols, and chalcones are all products of the flavonoid biosynthetic pathway. The flavonoid pathway is one of the best-characterized secondary metabolite pathways in plants, with all the major biosynthetic steps and many of the pathway regulatory genes defined for a range of model and crop species (Grotewold, 2006; Hichri et al., 2011; Davies et al., 2012a). The primary point of regulation for flavonoid biosynthesis commonly occurs at the transcriptional level. In all species studied to date, anthocyanin synthesis is regulated by a ‘MBW’ transcriptional activation complex consisting of R2R3-MYB and bHLH transcription factors (TFs) and a WD-repeat (WDR) protein, which acts directly upon the promoters of flavonoid biosynthetic genes (Ramsay and Glover, 2005; Feller et al., 2011; Hichri et al., 2011; Davies et al., 2012a). Distinct R2R3-MYBs regulate different branches of the flavonoid pathway, forming sub-groups (SG) within the large MYB family, such as anthocyanins (SG 5 and 6), proanthocyanidins (PAs, condensed tannins, SG5), flavonols (SG7), and isoflavonoids (SG2; Stracke et al., 2001; Feller et al., 2011; Shelton et al., 2012). Furthermore, the genes encoding the R2R3-MYB activators (which contain two repeats of a MYB DNA-binding domain) are thought to be key in determining the spatial and temporal occurrence of flavonoids in plants (Schwinn et al., 2006; Stracke et al., 2007; Allan et al., 2008; Feller et al., 2011; Davies et al., 2012a). Other TFs can have a repressive effect on phenylpropanoid production, either by interfering with the formation of the MBW activation complex or through formation of MBW complexes with an active repressive action (Davies et al., 2012a; Gray et al., 2012; Albert et al., 2014a).

The main models for studies on regulation of anthocyanin biosynthesis have been the monocot maize (*Zea mays*) and the dicots Arabidopsis (*Arabidopsis thaliana*), petunia (*Petunia hybrida*), and snapdragon (*Antirrhinum majus*), with crops such as apple (*Malus domestica*), grape (*Vitis vinifera*), and rice (*Oryza sativa*) being extensively studied more recently (Allan et al., 2008; Feller et al., 2011; Hichri et al., 2011; Gray et al., 2012). The regulatory system appears to be well conserved across the dicots and the monocots studied to date, but with divergence within the regulatory gene clades. While the separation into SG5 (PA-related) and SG6 (anthocyanin-related) is conserved in dicot species studied, the anthocyanin R2R3-MYB regulators identified from the Poaceae and Orchidaceae cluster in SG5 (Davies et al., 2012a). To date, anthocyanin-related SG6 R2R3-MYB genes from monocot species have been characterized only in Asiatic lily (*Lilium* spp., belonging to the order Liliales; Yamagishi et al., 2010). Sequences for other flavonoid-related MYB SG have not so far been characterized from non-Poaceae monocot species. In particular, genes that regulate flavonoid synthesis have not yet been identified in onion, although it has been suggested that the classical *C* locus may correspond to a regulatory gene (Kim et al., 2005b). In this investigation, we identify a range of R2R3-MYB factors that putatively regulate flavonoid production in onion. One, MYB1 is characterized as a key positive regulator of anthocyanin production.

## Materials and Methods

### Isolation of cDNA and Genomic DNA Sequences

Pieces of acyanic internal sheaths from seedlings of onion ‘California Early Red’ were cultured on 1/2 MS medium under 20–50 μmol m^-^2 s^-^1 light from Osram 36 W Grolux fluorescent tubes (16 h photoperiod) at 25°C. Under these conditions, anthocyanin production was sporadically initiated by 24 h and increased over 96 h. *MYB1* was isolated from tissue at 48 h using 3′- and 5′-RACE (Invitrogen 5′-RACE system). Genomic regions corresponding to the *MYB1* cDNA sequence were isolated using PCR with primers designed to the cDNA. A genomic region upstream of the coding sequence was obtained from three overlapping PCR products using the GenomeWalker^TM^kit (Clonetech, Mountain View, CA, USA). Primer sequences are given in Supplementary Table 1.

SG4 (MYB4, MYB5) gene fragments were first isolated by 3′-RACE as described for *MYB1*, with full-length sequences subsequently identified from a transcriptome database generated using TGICL software as described in Crowhurst et al. (2008) from sequences from the following sources: GenBank NT division mRNA sequences, Onion ESTs (Kuhl et al., 2004), RNASEQ data from NCBI BioProjects PRJNA175446 and PRJNA175449 (Duangjit et al., 2013), and PRJNA238142 and PRJNA60277 (Baldwin et al., 2012). The SG7 (MYB29) sequence also was identified from the transcriptome database. Primer sequences are given in Supplementary Table 1. The onion MYB sequences were assigned numbers to reflect the order in which they were identified, as per the guidelines of Gray et al. (2009).

### Phylogenetic and Promoter Sequence Analysis

Maximum likelihood phylogenetic trees based on full-length sequences were generated using PhyML with 1000 bootstrap replicates (Guindon and Gascuel, 2003) upon MUSCLE (Edgar, 2004) amino acid alignments (with manual correction) within Geneious (v7). Analysis of the putative TF recognition sites in the promoter regions used the PLACE database (Higo et al., 1999). GenBank database accession numbers are *Allium cepa* MYB1 (KX785130), MYB4 (KX785131), MYB5 (KX785132), MYB29 (KX785133); *Antirrhinum majus* ROSEA1 (ABB83826), MIXTA (CAA55725), MYB308 (P81393); *Aquilegia formosa* MYB17 (ACQ82820); *Arabidopsis thaliana, MYB4 (At4g38620), MYB12 (ABB03913), MYB20 (NP_176797), MYB24 (AF175987), MYB75/PAP1 (AAG42001), MYB79 (AEE83284), MYB103 (AED96722), MYB106 (AEE73615), MYB123/TT2 (CAC40021); Dahlia pinnata* (syn. *variabilis*) MYB1 (AB601003); *Gerbera hybrida* MYB1 (CAD87007), GMYB10 (CAD87010); *Glycine max* MYBZ2 (LOC780553); *Gynura bicolor* MYB1 (BAJ17661); *Ipomoea nil* MYB1 (BAE94391); *Lilium* (hybrid division I) MYB6 (BAJ05399), MYB12 (BAJ05398); *Lotus japonicus* TT2a (AB300033); *Malus* x *domestica* MYB10 (ACQ45201), MYB16 (ADL36756); *Medicago truncatula* LAP1 (ACN79541); *Oncidium* Gower Ramsey MYB1 (ABS58501); *Oryza sativa* C1 (BAD04024), IF35 (BAB20661), Plw-OSB1 (BAB64301); *Petunia* x *hybrida* AN2 (AAF66727), PH4 (ADX33331); *Phalaenopsis schilleriana* UMyb6 (FJ039860); *Picea mariana* MBF1 (U39448); *Vanda hybrida* MYB6 (ADQ57817); *Vitis vinifera* MYBA1 (BAD18977), MYB4a (ABL61515), MYBPA1 (AM259485), MYBF1 (FJ948477); *Zea mays* C1 (AAA33482), P1 (AAC49394), MYB38 (P20025).

### Analysis of Transcript Abundance Using qPCR

Quantitative RT-PCR (qPCR) was used to measure transcript abundance for *MYB1, CHS-A*, and *DFR-A* in white and red colored regions of onion seedlings. *DFR-A* (AY221249) is the only significantly active DFR gene in the onion bulb (Kim et al., 2004a) and *CHS-A* (AY221244) is one of the two significantly active CHS genes (Kim et al., 2005b). Outer leaves of red onion seedlings were removed to expose the first fully white sheath beneath. Each seedling was cut in half longitudinally and the white sheath tissue of one half was immediately flash frozen in liquid N_2_. The other half was placed on moistened paper in a Petri dish and left to become pigmented. Pigmented sheath tissue was sampled after approximately 3 days. Two samples were analyzed, each consisting of sheath tissue from three seedlings. Total RNA was isolated using the RNeasy Plant Mini Kit (Qiagen). First strand cDNA was made from 500 ng DNAse I-treated RNA using the Transcriptor First Strand cDNA Synthesis Kit (Roche) and priming from the supplied anchored oligo dT_18_. qPCR (on cDNA diluted 50-fold) was performed as described in Albert et al. (2011). Relative transcript abundance was determined by comparative quantification. Primers used are given in Supplementary Table 1.

### Particle Bombardment Experiments

Overexpression vectors for *MYB1* (pKES22) and the maize anthocyanin bHLH factor *Lc* (pNASA3) were generated by cloning the respective cDNAs behind a *CaMV 35S* promoter (*35Spro*) in pART7. The RNAi vector for *MYB1*, pHMN3, was based on pDAH2 (Davies et al., 2012b). The construct, driven by *35Spro*, had inverted repeats to give hairpin RNA. Primers used in making these constructs are given in Supplementary Table 1. Other constructs used were for a GFP internal transformation control (pPN93; *35Spro:GFP-ER*; Shang et al., 2007), a snapdragon anthocyanin bHLH factor (pJAM1528; *2x35Spro:AmMutabilis* in pJIT60; supplied by Cathie Martin) and a maize anthocyanin MYB factor (pLN44; *35Spro:ZmC1* in pART7; Albert et al., 2010). Constructs were transformed into dorsal petals of the snapdragon *rosea^dorsea^* mutant line (Schwinn et al., 2006), seedling tissue of onion ‘California Red,’ and shoot tissue from bulbs of an unidentified commercial cultivar of garlic (*Allium sativum*). Particle bombardment using a helium-driven particle in flow gun was carried out generally as described in Davies et al. (2012b). Tissue was not surface sterilized before bombardment. Each experiment involved the bombardment of at least three pieces of tissue with each piece bombarded once (onion/garlic) or twice (snapdragon). Each experiment was replicated at least twice except for the experiment involving pJAM1528. Bombardment used a 30 ms burst of helium at a pressure of 200 kPa for the RNAi experiments, 300 kPa for the other experiments in onion and garlic, and 400 kPa for the experiments in snapdragon. DNA concentrations in the gold particle preparations were 0.4 μg/1 mg gold for the GFP construct, which was added to all preparations, and either 0.8 or 1.0 μg/1 mg gold for each of the other constructs added to a preparation. Post-bombardment onion tissue was placed on moistened filter paper in a Petri dish. Images were recorded using a Leica M205FA microscope with a Leica DFC550 digital camera or an Olympus SZX fluorescent microscope (Olympus Corp., Tokyo, Japan) with a Leica DC500 digital camera (Leica Camera AG, Solms, Germany). Any post image-capture adjustments of brightness or contrast for clarity of the GFP signal were applied equally to all images in a figure.

### *Agrobacterium*-Mediated Transformation of *Allium sativum*

Transgenic garlic plants (‘Printanor’) were produced using the method of Kenel et al. (2010). Cassettes for both *35S:MYB1* and *35S:ZmLc* were introduced into pART27H, a modified version of pART27 (Gleave, 1992) that has the *NPTII* selectable marker replaced with an *HPTII* (hygromycin) expression cassette. A schematic of the plasmid is shown in Supplementary Figure 2. Presence of the selection marker *HPTII* was detected using PCR on genomic DNA isolated from tissue culture leaf tissue.

## Results

### Identification of a Candidate Anthocyanin-Related R2R3-MYB Factor

Candidate *R2R3-MYB* genes for anthocyanin regulation were amplified using 3′-RACE PCR from cDNA derived from RNA purified from onion tissue that was induced to produce anthocyanins (see “Materials and Methods”). Degenerate oligonucleotides designed to the conserved MYB DNA-binding domain of R2R3-MYB genes were used as forward primers (Supplementary Table 1). Several *MYB*-related sequences were obtained, including one sequence with similarity to anthocyanin-related SG6 MYB genes. The partial cDNA sequence for this candidate gene was extended by 5′-RACE, followed by PCR amplification of a full cDNA coding sequence. This gene was named MYB1 (GenBank accession KX785130). The *MYB1* cDNA is 1036 bp long with three possible ATG translation initiation codons, all of which are in frame from the first ATG. The second of these has the best fit to the Kozak consensus sequence, and on this basis the deduced polypeptide sequence consisted of 253 amino acids. The closest matches to MYB1 on a BLASTp search of the NCBI non-redundant protein sequence database were anthocyanin-related R2R3-MYB proteins, but from dicot rather than monocot species (data not shown).

Based on data from other species it was expected that a small multigene family of anthocyanin-related R2R3-MYB activators would be present in onion. A second 3′-RACE sequence was isolated that was identical to MYB1 except for possessing a 92 bp direct repeat in the coding sequence, and a truncated 3′-UTR (21 bp in length) containing one single nucleotide polymorphism. Compared with MYB1, the direct repeat altered the predicted polypeptide sequence, causing 10 sequential variant amino acids followed by a truncation of 75 amino acids in the C-terminus due to a premature stop codon (data not shown). It is not known if this cDNA represents an allele of MYB1. To look for further *MYB1* related alleles or genes, a subsequently available in-house transcriptome database was searched (BLASTn and BLASTp) with the *MYB1* sequence. The transcriptome examined contained approximately 296, 942 singleton and contig assemblies from sequencing 13 different onion mRNA derived libraries. No additional anthocyanin-related *MYB* genes were detected. However, four partial sequences (between 225 and 615 bp) were identified that differed from *MYB1* by less than 10 nucleotides and which probably represent either allelic differences or sequencing errors (data not shown).

**FIGURE 1:**
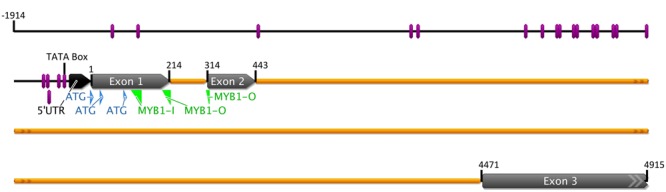
**Graphic representation of the *MYB1* gene of onion (*Allium cepa*).** Exons, introns, 5′-UTR, the three potential ATGs, and the putative TATA box are marked. Positions of notable sequence motifs for transcriptional regulation identified using the PLACE website analysis are shown by the purple bars. Locations of DNA primers used for genome walking for promoter isolation are indicated in green. The upstream genomic sequence annotated for the specific DNA-binding motifs is given in Supplementary Figure 1.

The genomic *MYB1* sequence (from the ATG furthest upstream through the stop codon) was isolated from the same onion cultivar used to isolate the full cDNA. It was 4.915 kb in length and the gene contained three exons and two introns (intron 1 is 0.099 kb and intron 2 is 4.027 kb; **Figure 1**), a common structure in plant *R2R3-MYB* genes. In addition a total of 1.914 kb upstream of the distal start codon was isolated by genome walking. To understand how this gene may be regulated in response to light and other factors, the PLACE database for predicted DNA-binding recognition sites (Higo et al., 1999) was used to analyze the proximal 1.0 kb of this upstream region. A predicted TATA box was located at -25 bp. There was a cluster of predicted recognition motifs of interest within the -500 bp region, including candidate MYB and bHLH sites and a range of sites found in promoters of genes up-regulated by light, such as the T-Box, GT-1, and SORLIP1AT motifs (**Figure 1**; Supplementary Figure 1).

### Identification of Candidates for Other Flavonoid-Related R2R3-MYB Factors

Flavonols are other key flavonoid metabolites in onion bulbs. Given that R2R3-MYB proteins of SG7 have been found to regulate the production of flavonols in a range of dicot species and in the monocot maize, we reasoned that SG7 MYB genes regulate flavonol production in onion. Several highly similar sequences were retrieved from a transcriptome database when searched (BLASTp) with the sequence for the flavonol regulator At-MYB12 from Arabidopsis, including a singleton sequence encoding a full-length candidate, *MYB29* (GenBank accession KX785133). The *MYB29* sequence was 1076 bp long, with a putative 5′- and 3′-UTR of 19 and 187 bp, respectively. The EST originated from a mixed tissue mRNA sample of the doubled-haploid red onion genotype 5225. Analysis of related sequences identified one additional SG7 gene family member in the data from the doubled-haploid line, represented by a partial 5′-EST (data not shown).

The deduced amino acid sequence of MYB29 was used to search (BLASTp) the NCBI non-redundant protein sequence database. The closest matches to MYB29 were At-MYB12 and another SG7 protein, Zm-P1, a regulator of flavone and phlobaphene production in maize. The motif identified as conserved among flavonol-related SG7 R2R3-MYBs ([K/R][R/x][R/K]xGRT[S/x][R/G]xx[M/x]K; Stracke et al., 2001) was fully conserved in MYB29 as KKRKGRTSRSAMK.

The initial 3′-RACE screen for anthocyanin MYBs predominantly isolated R2R3-MYB sequences belonging to SG4. Characterized SG4 sequences from other species encode proteins with a repressive action on genes of the phenylpropanoid pathway. Full-length cDNAs for two of the genes from the screen, *MYB4* and *MYB5* (GenBank accessions KX785131 and KX785132, respectively), were subsequently identified in the transcriptome database. The *MYB4* sequence was 844 bp long with a deduced peptide of 213 amino acids, and matched a partial EST present on GenBank (CF441129). *MYB5* was 1144 bp in length with a deduced peptide of 236 amino acids. The closest matches to MYB4 or MYB5 on a BLASTp search of the GenBank Swiss-Prot database were Am-MYB308 from snapdragon and At-MYB4 from Arabidopsis. As with other members of SG4, MYB4, and MYB5 contain in their C-terminals an ERF-associated Amphiphilic Repression (EAR) domain (C2 motif) and the lsrGIDPxT/NHR (C1) motif as defined by Kranz et al. (1998) (Supplementary Figure 2).

### Phylogenetic Analysis of the Candidate Flavonoid Regulators

**FIGURE 2:**
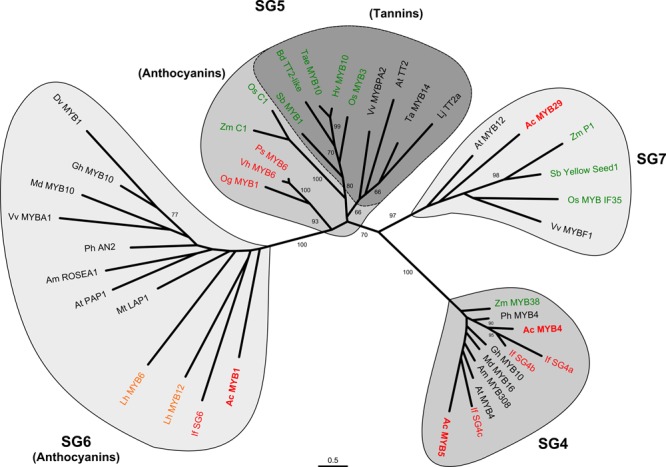
**Phylogenetic relationships of onion (*Allium cepa*) flavonoid-related transcription factors to those of other species.** Full-length deduced amino acid sequences, Ac-MYB1, Ac-MYB4, Ac-MYB5, and Ac-MYB29 were used to form phylogenetic trees with selected flavonoid-related transcription factors from other plant species. The regulators of the different flavonoid types form separate sub-groups (SGs), as indicated, with a further division of anthocyanin regulators into two subgroups (SG5 and SG6 as indicated by TT2 and PAP1 of *A. thaliana*, respectively). GenBank accession numbers of the sequences used are listed in “Materials and Methods.” Bootstrap values >60% are shown (1000 replicates). Asparagales, Liliales, or Poaceae species are shown in red, orange, and green, respectively.

The relationships of MYB1, MYB4, MYB5, and MYB29 to a range of characterized, flavonoid-related R2R3-MYBs were examined by phylogenetic analysis (**Figure 2**). The candidate onion sequences grouped with the expected flavonoid regulators. Specifically, MYB29 grouped with SG7 (flavonol/flavone-related) factors, while MYB4 and MYB5 grouped with SG4 (repressors of flavonoid biosynthesis) factors, although in different subclades within SG4. MYB5 showed some amino acid residue changes within the conserved MYB domain as well as some variation in other C-terminal motifs that have been categorized by Cavallini et al. (2015) (Supplementary Figure 2).

There are two clades within the anthocyanin-related R2R3-MYBs, SG5, and S6, based on sequences that are currently available (Yamagishi et al., 2010; Feller et al., 2011; Davies et al., 2012a). These may be defined as whether sequences separate with Arabidopsis At-MYB75 (PAP1) or At-MYB123 (TT2), which regulate anthocyanin and proanthocyanidins (PAs or condensed tannins), respectively. MYB1 grouped with anthocyanin-related factors in SG6, including ones from another Asparagales taxon (*Iris fulva*; If-SG6) and a taxon in the monocot order Liliales (*Lilium hybrida*, Asiatic lily; Lh-MYB6, Lh-MYB12). This contrasts with the anthocyanin-regulating R2R3-MYBs from orchid taxa (Og-MYB1, Vh-MYB6, Ps-MYB6), which despite being members of the Asparagales, are located within SG5 along with representative sequences from the monocot order Poales.

Alignment of a region of 104 amino acid residues containing the MYB domain of SG5 and SG6 sequences (including MYB1) identified five conserved amino acid or motif differences among the clades (Supplementary Figure 3). These were [R] versus [N/H/K], [C/F] versus Y, R versus G, X versus N, and A versus D for the SG6/PAP1 versus SG5/C1-TT2 clade, respectively. These differences are strongly conserved within each clade and are a likely cause of the clade separation. Notably, the A versus D (position 90 in Supplementary Figure 3) was the start of a DNEI motif that is absolutely conserved in the SG5 sequences (and other subgroups like SG4 and SG7) but absent in the SG6 sequences, where the motif was ANDV in all but one example. Both SG5 and SG6 proteins (including MYB1) contain a conserved motif ([D/E]Lx_2_[R/K]x_3_Lx_6_Lx_3_R) within the MYB domain that is necessary to bind bHLH partners (Zimmermann et al., 2004), and is an essential component to the formation of MBW activation complexes.

**FIGURE 3:**
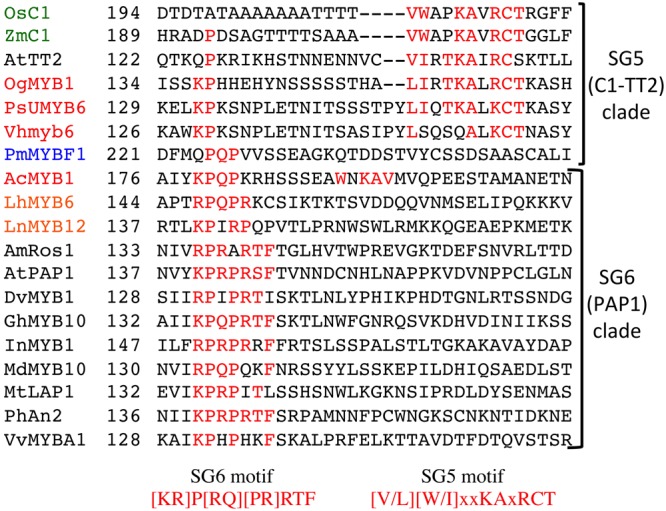
**Alignment of part of the C-terminal region of R2R3-MYB sequences showing the SG5 and SG6 motifs.** Amino acids matching either the SG6 motif or the SG5 (C1-TT2) clade motif are indicated in red. Species names are as given in the “Materials and Methods,” with Asparagales, Liliales, Poaceae, Gymnospermae, and dicot species shown in red, orange, green, blue, and black, respectively. The starting amino acid position of the sequences is given in the second column.

Within the variable C-terminal region, a motif has been identified as defining the anthocyanin-related SG6 proteins. This was initially identified as KPRPR[S/T]F when SG assignments were made for Arabidopsis (Stracke et al., 2001), and later modified to [K/R]P[Q/R]P[Q/R] based on the Asiatic lily SG6 sequences (Yamagishi et al., 2010). This motif was reasonably conserved within MYB1, as KPQPxxx, but is very poorly conserved in the Orchidaceae and Poaceae sequences that form the C1-TT2 clade (**Figure 3**). A second motif, closer to the C-terminal, was conserved within the C1-TT2 clade, although it aligned less consistently than the previously defined KPRPR[S/T]F. This is VWAPKAVRCT in C1 and VIRTKAIRCS in TT2, and it was strongly conserved except in Vh-MYB6 (**Figure 3**). The V[V/I]RTKA sequence is strongly conserved in a range of R2R3-MYBs involved in activation of PA production (Liu et al., 2014).

### *MYB1* Transcript Abundance Correlates with Anthocyanin Production

**FIGURE 4:**
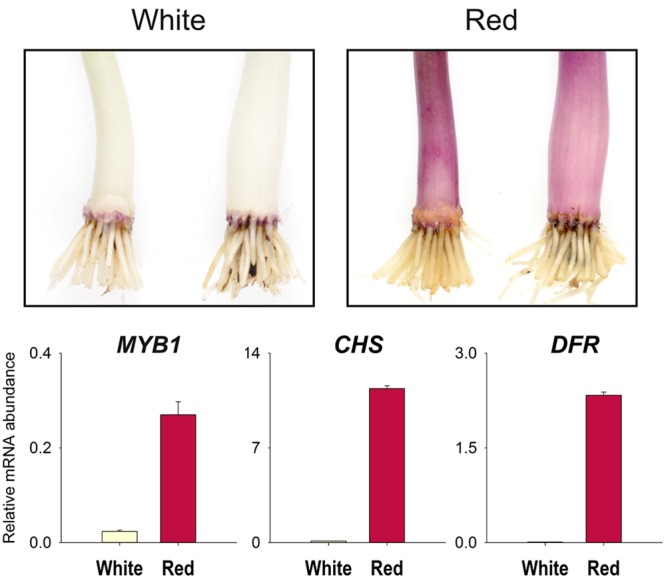
**Increased *MYB1* transcript abundance is associated with anthocyanin production.** Quantitative RT-PCR (qPCR) analysis of red onion (*Allium cepa*) seedling leaf sheath tissue for expression of *MYB1* and two anthocyanin biosynthetic genes, *chalcone synthase-A* (*CHS*) and *dihydroflavonol 4-reductase-A* (*DFR*). Transcript abundance was normalized to the geometric mean of the transcript quantities of *UBQ* and *GAPDH*. The sample consists of pooled tissue from three seedlings. Means ± SEM, *n* = 3 technical replicates are shown. A replicate qPCR experiment gave results consistent with the data presented. Top panels illustrate the phenotype and the type of tissue analyzed rather than an actual sample.

The spatial and temporal expression patterns of the R2R3-MYB genes have been shown to be important in determining anthocyanin phenotypes in a range of species. Transcript abundance for *MYB1* and two anthocyanin biosynthetic genes, *chalcone synthase* (*CHS*) and *DFR*, was compared between pooled samples of white sheaths and sheaths in which anthocyanin production was induced. Transcript amounts for *MYB1, CHS*, and *DFR* were much higher in the scales that had become pigmented (**Figure 4**).

### Transient Overexpression of *MYB1* Induces Anthocyanin Production

**FIGURE 5:**
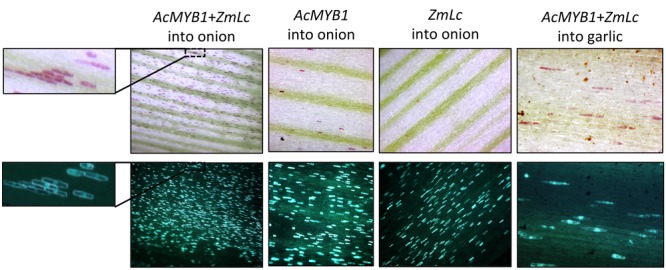
**Transient overexpression of *MYB1* induces anthocyanin pigmentation in onion (*Allium cepa*) and garlic (*Allium sativum*).** Tissue was bombarded with constructs for *35S:MYB1*+*35S:Zm-Lc*+*35S:GFP, 35S:MYB1*+*35S:GFP*, or *35S:Zm-Lc*+*35S:GFP*. Tissue from acyanic red onion seedling leaf sheaths and garlic cloves was used. Images are shown under white light **(Top panels)** for anthocyanin pigmentation or blue light **(Lower panels)** for GFP fluorescence. A close up of one region is shown for easier comparison of the co-localisation of GFP signal and red pigmentation. Representative images are shown.

The ability for MYB1 to activate anthocyanin synthesis was tested in both onion and garlic using biolistic transformation (**Figure 5**; Supplementary Figure 4). Introduction of *35S:MYB1* into acyanic sheath tissue of inner leaves or inner sprouts of red onion seedlings resulted in single cell red foci at low frequency that were visible by 48 h post-bombardment. However, co-bombardment of *35S:MYB1* with constructs for heterologous anthocyanin bHLH regulators (*Zm-Lc* and *Am-Mutabilis*) resulted in a greater frequency of red cells (**Figure 5**; Supplementary Figure 4) and these became visible earlier. Particularly in green sprout tissue, the red cells were visible within 12–24 h post-bombardment. The anthocyanin pigmentation co-localized with GFP fluorescence, further indicating that the pigmentation was due to introduction of the TFs and not from autonomous activation of pigmentation. Autonomous activation does occur, but its delayed timing allowed sufficient time to conduct the experiments. Introduction of the bHLH factor alone did not induce anthocyanin production, nor, interestingly, did the introduction of the SG5 anthocyanin regulator from maize, *ZmC1*, in conjunction with *Zm-Lc* (Supplementary Figure 4). Anthocyanin synthesis was also activated in garlic shoot tissue after co-bombardment with *MYB1* and bHLH (*Zm-Lc*) constructs (**Figure 5**), but not when *MYB1* alone was used.

### Transient RNAi Inhibition of *MYB1* Prevents Anthocyanin Formation

**FIGURE 6:**
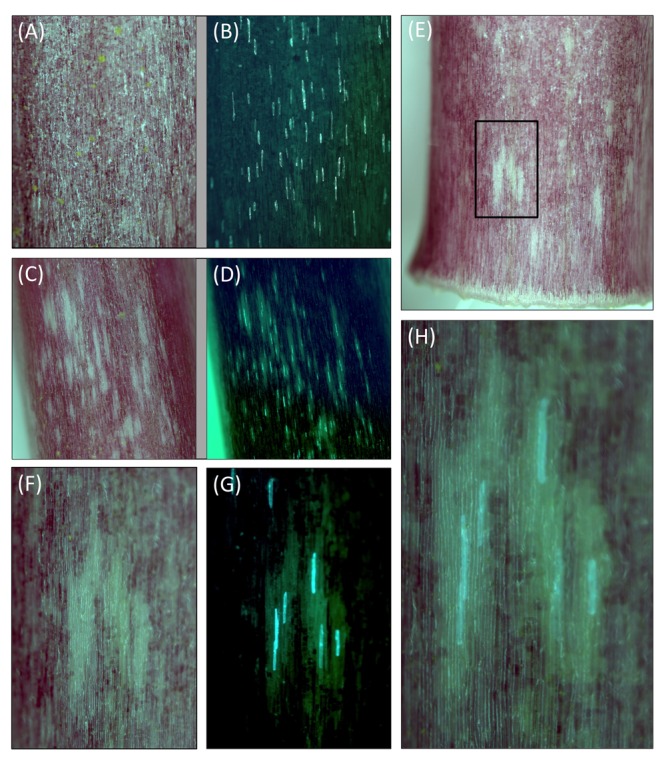
**Transient RNAi inhibition of *MYB1* expression inhibits anthocyanin pigment formation in onion (*Allium cepa*) seedlings.** Acyanic sheath tissue was removed from inner layers of red onion seedlings and biolistically transformed with gene constructs for *35S:GFP* and *35S:MYB1-RNAi* (two different example regions are presented in **C–H**) or *35S:GFP* alone (example region in **A,B**). They were then incubated for around 72 h on moistened filter paper in a Petri dish and exposed to a 16:8 h light:dark cycle. White regions, lacking anthocyanin pigmentation, were observed only when *35S:MYB1-RNAi* was used. **(B,D,G)** Are blue light images for GFP visualization. **(F,G)** Show a close up of one white region (boxed in **E**), and **(H)** shows a further close up in which a partially transparent version of the white light image for anthocyanins (F) is overlaid on the GFP image (H). Representative images are shown.

To investigate further whether *MYB1* was a key gene determining anthocyanin pigmentation in onion, transient RNAi was conducted using biolistic introduction of a hairpin construct for *MYB1*. The autonomous induction of anthocyanin production in seedling leaf sheath tissue described earlier allowed the effect of RNAi against *MYB1* to be observed. Acyanic sheath tissue was bombarded with the *35S:MYB1-RNAi* and *35S:GFP* constructs after it was removed from red onion seedlings, and incubated to allow pigmentation to develop. In tissue bombarded with the MYB1 RNAi construct, zones with greatly reduced or absent pigmentation became visible by 48 h post-bombardment and by 72 h they were more prominent, as the pigmentation in the background of untransformed cells developed (**Figure 6**). As expected for RNAi, the inhibition was not cell autonomous, but rather spread to adjacent cells, as indicated by multi-cellular acyanic regions surrounding the central transformed cells (GFP positive cells). Bombardment with *35S:GFP* alone had no visible effect on the anthocyanin phenotype (**Figure 6**).

### Ectopic Red Pigmentation in Garlic Transformed with *MYB1* and Maize *Lc*

**FIGURE 7:**
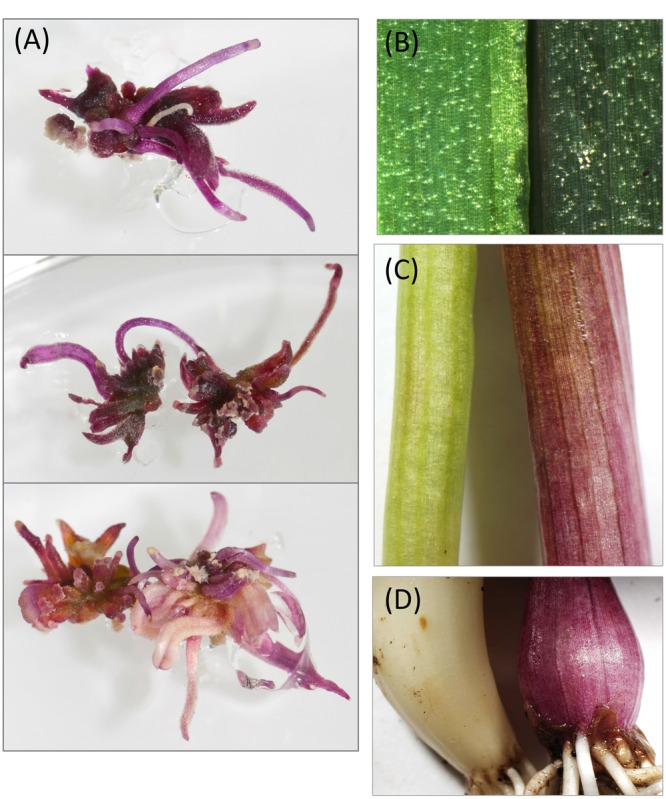
**Phenotypes of garlic (*Allium sativum*) plants stably transformed with a construct for *35S:MYB1* and *35S:ZmLc*. (A)** shows examples of regenerating shoots of independent transgenic events in culture. **(B–D)** show tissue from, respectively, the leaf, stem base, and bulb, with the *MYB1+Lc* transgenic (line 1111-8A) on the right and a control plant on the left. The control was a transgenic carrying a construct unrelated to pigmentation (an antiviral construct).

Garlic rather than onion was chosen for this experiment because it is technically easier to transform. To maximize potential for anthocyanin production in the transgenic plants, garlic was transformed via *Agrobacterium* with both *35S:MYB1* and *35S:Zm-Lc* transgenes. The transgenes were present on the same T-DNA, in a binary vector containing a hygromycin selectable marker (Supplementary Figure 5). Strong red pigmentation was present on the callus, and this was visible from early in the transformation/regeneration process (**Figure 7**). This phenotype has not been observed when using other gene constructs with the same tissues and methods (e.g., Kenel et al., 2010). The young regenerated transgenic plants had darker pigmentation of the leaves than control plants and red pigmentation at the base of the leaves, where the bulb would develop. Four independent transformation events were taken through to mature plants (1111-1A, 1111-3A, 1111-7A, 1111-8A). These showed variable intensity of novel pigmentation, including light-pink (1111-3A) and strong red (1111-8A). The plants were maintained through a season of bulb formation, plant dieback and dormancy, and bulb sprouting in the following spring. The newly formed foliage continued to show darker pigmentation of the leaves and red pigmentation at leaf bases (**Figure 7**).

### *MYB1* Can Substitute for a Dicot R2R3 Anthocyanin Regulator

**FIGURE 8:**
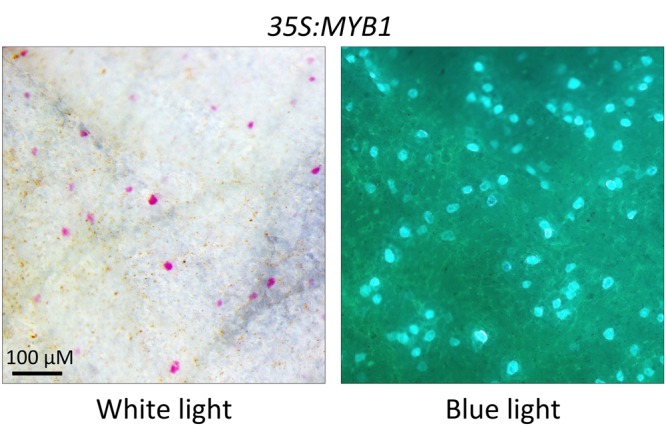
**Complementation of the white petal phenotype of the *Antirrhinum majus* R2R3-MYB mutant *rosea^dorsea^* by *MYB1*.** Representative image shown of cell autonomous anthocyanin production after bombarding *35S:MYB1+35S:GFP* into the inner epidermis of petal tissue. Anthocyanin production co-localizes with GFP expression. Images were photographed under white light for pigmentation and blue light for GFP fluorescence.

Given that MYB1 is phylogenetically more similar to dicot anthocyanin regulators than those from orchid or grasses (i.e., from SG5), we tested whether it could substitute for a dicot regulator. Along with a GFP internal control, the *35S:MYB1* construct was biolistically introduced into acyanic petal tissue of the *rosea^dorsea^* anthocyanin *MYB* mutant of snapdragon (Schwinn et al., 2006). MYB1 was able to complement the mutant, resulting in cell autonomous production of anthocyanin pigment, which co-localized with GFP fluorescence (**Figure 8**). Introduction of a reporter gene alone does not restore pigmentation in this mutant (Schwinn et al., 2006).

## Discussion

Flavonoids are a key group of secondary metabolites in plants, with roles ranging from attraction of pollinators to defense against abiotic and biotic stresses. Their production is principally regulated through alterations in the transcription rate of their biosynthetic genes, and the pathway is one of the main model systems for understanding transcriptional regulation in plants. There are several well-established dicot model species, as well as the monocot maize, for the study of the flavonoid pathway. Apart from maize, there is only limited information on the regulation of flavonoid biosynthesis in monocot crop species, and that is mostly from two other Poaceae species – rice and *Sorghum*. Onion is a major crop species from the largest monocot order, the Asparagales, which contains around 29,000 species. Flavonoids are key to the visual appeal and human health characteristics of onion. However, while there has been much research on the genetic loci and biosynthetic genes underlying flavonoid production in onion, there is little information about the regulatory genes for the pathway. In this study, we have identified five flavonoid-related TF sequences from onion, characterized the importance of the R2R3-MYB factor MYB1 in controlling anthocyanin biosynthesis, and demonstrated the utility of MYB1 for application in the molecular breeding of *Allium* crops.

### *MYB1* Regulates Anthocyanin Synthesis

A candidate gene approach was taken to identify genes that regulate the production of anthocyanin pigments in onion. *MYB1*, a sequence sharing similarity to characterized anthocyanin-related MYB genes, was identified from red onion. In these onions, anthocyanin pigmentation is first present in the seedlings. It occurs in the swelling, predominantly achlorophyllous sheath tissue of the first few outer layers of the leaves. As the bulb develops and matures, pigmentation extends throughout the inner layers, comprised of the swollen sheaths and scales. *MYB1* expression (along with expression of the investigated biosynthetic genes, *CHS* and *DFR*) was correlated with anthocyanin induction in sheath tissue of the seedlings (**Figure 4**). Furthermore transient overexpression and knockdown experiments induced and inhibited, respectively, anthocyanin production in the seedlings (**Figures 5** and **6**), and ectopic anthocyanin production was observed in garlic co-transformed with transgenes encoding MYB1 and a heterologous bHLH factor (ZmLc; **Figure 7**). These results demonstrate that MYB1 positively regulates anthocyanin pigmentation and we conclude that MYB1 is key for the red bulb phenotype of this crop. As such it is an excellent candidate for underlying a major Quantitative Trait Locus (QTL) controlling anthocyanin concentration and intensity of red bulb color. Duangjit et al. (2014) have identified two such QTLs that are different from the regions controlling red versus yellow bulbs that have been attributed to the *L* locus (which encodes the anthocyanin biosynthetic gene *ANS*) by Kim et al. (2006). They proposed that these newly characterized QTLs may be associated with factors that regulate the pigment pathway.

Vegetative tissues of plants often accumulate anthocyanins in response to light (Gavazzi et al., 1990; Dubos et al., 2008; Albert et al., 2009), particularly in juvenile tissues that may be more sensitive to excess irradiation (Gould, 2004). In onion seedlings only a few of the outer layers of leaves showed anthocyanin pigmentation in the sheaths and when these were peeled away, the exposed acyanic sheath tissue beneath rapidly became pigmented in our experimental conditions. This suggests that light is an important signal in anthocyanin production in onion, at least early in plant development. This signal is probably integrated through control of MYB1. The promoter region of *MYB1* has recognition motifs that have been observed in light regulated genes (Supplementary Figure 1). In addition, the induced accumulation of anthocyanins in acyanic inner leaf sheaths was correlated with the expression of *MYB1* (**Figure 4**). Juvenile shoots of Asiatic lily produce anthocyanins upon exposure to light because of the expression of the anthocyanin regulators *Lh-MYB6, Lh-bHLH1*, and *Lh-bHLH2* (Nakatsuka et al., 2009; Yamagishi et al., 2010). Therefore, light-controlled anthocyanin synthesis is probably regulated by a similar mechanism in both lily and onion. Other signals are expected to be involved in the regulation of anthocyanin production in red onion since by the time the bulbs mature the inner swollen sheaths and scales, all substantially thickened tissues, have become pigmented without being directly exposed to light. It is possible that a light signal is being transduced to the inner leaves; however, pigment regulation during bulb development is likely to involve developmental signals as well.

### MBW Complex Indicated for Regulation of Anthocyanin in *Allium*

A MBW complex regulates anthocyanin synthesis in all species studied to date. While most of the functional data for the MBW complex are from eudicots, the cooperative involvement of MYB, bHLH, and WDR proteins for regulating anthocyanin synthesis is well-established in the monocot model maize (Ramsay and Glover, 2005; Feller et al., 2011). The predicted amino acid sequence of MYB1 has the motif necessary to bind bHLH TF partners (Supplementary Figure 3), and the co-introduction of a bHLH factor with MYB1 in the transient assays allowed earlier development of anthocyanin pigmentation along with an increased frequency of pigmented cells (**Figure 5**). MYB1 was able also to substitute for the endogenous R2R3-MYB anthocyanin regulator in a dicot species that is a model for MBW regulation of anthocyanin pigmentation (Schwinn et al., 2006) (**Figure 8**). In the orchid family (also Asparagales), anthocyanin synthesis is also activated when R2R3-MYB and bHLH proteins are co-expressed (Ma et al., 2008; Albert et al., 2010), and endogenous R2R3-MYB genes that regulate anthocyanin production have been identified in the orchid genera *Phalaenopsis* (Ma et al., 2009) and *Oncidium* (Chiou and Yeh, 2008). This suggests that the MBW complex is conserved within Angiosperms, although some aspects of MBW activity have yet to be identified in monocots (Albert et al., 2014b). These include hierarchical activation of bHLH proteins, the involvement of gene-regulation networks, and the action of repressor proteins, which are conserved in eudicots (Albert et al., 2014a). In many species, the IN/AN1/MUT-clade bHLH is essential for regulating anthocyanin synthesis, even if LC/JAF13/DEL-clade bHLH proteins are expressed (Davies et al., 2012a). In Asiatic lilies, the anthocyanin-related R2R3-MYBs (Lh-MYB6 and Lh-MYB12) were able to activate the promoters of the *Lh-CHSa* and *Lh-DFR* genes only when Lh-bHLH2 (IN/AN1/MUT clade) was co-bombarded (Yamagishi et al., 2010), although Lh-bHLH1 (LC/JAF13/DEL) was not tested. In maize, the LC/JAF13/DEL clade bHLH factors regulate anthocyanin synthesis (Ludwig et al., 1989; Tonelli et al., 1991, 1994; Goff et al., 1992), but the IN/AN1/MUT-clade bHLH has lost the ability to activate transcription and acts as a competitive repressor (Burr et al., 1996). Similar patterns of anthocyanin induction were observed in the onion transient assays when bHLH factors of either clade (ZmLC of the LC/JAF13/DEL clade and AmMUT of the IN/AN1/MUT clade) were co-introduced with MYB1 (**Figure 5**; Supplementary Figure 4). This indicates either that MYB1 normally interacts with bHLH factors of both clades in its native environment or that it has retained the ability to function with either type. Identification of the bHLH factors important for pigmentation in *Allium* is required to investigate this further.

### Identification of SG4 and SG7 R2R3-MYB Sequences from Onion

Flavonols accumulate to high concentrations in the bulbs of yellow and red onions, but are only present in trace amounts in white varieties (Patil et al., 1995). MYB29 was identified that has significant similarity to SG7 R2R3-MYBs, including the characteristic motif present in the C-terminus. While functional data are desirable, the phylogenetic support for Ac-MYB29 belonging to SG7 is very strong. In Arabidopsis, grape and tomato, the SG7 flavonol regulators coordinately regulate the early flavonoid genes *CHS, CHI*, flavanone 3-hydroxylase (*F3H)*, and the flavonol-specific gene flavonol synthase (*FLS*; Mehrtens et al., 2005; Luo et al., 2008; Czemmel et al., 2009; Ballester et al., 2010; Stracke et al., 2010). Similarly in maize, P1 directly regulates the *FLS* genes (Ferreyra et al., 2010, 2012), although P1 also regulates flavone and phlobaphene synthesis in some tissues (Casati and Walbot, 2005; Grotewold, 2006; Morohashi et al., 2012). However, it is also worth noting that in maize, *FLS* is also regulated by the anthocyanin-related MYB and bHLH factors (Ferreyra et al., 2010), suggesting a degree of functional redundancy exists. Taking this into consideration may help to explain the nature of the *C* locus in onion, which is thought to encode a regulatory gene. *C* is required for the expression of two genes encoding CHS (Kim et al., 2005b), which is the first committed step toward the biosynthesis of flavonoids, including flavonols and anthocyanins. MYB29 and MYB1 are excellent candidates for regulating *CHS*, but it is not currently known if they co-localize to the *C* locus, which is on chromosome 6 (Khar et al., 2008).

Onion R2R3-MYB DNA sequences were also isolated that belong to the phylogenetic SG containing repressors of phenylpropanoid/flavonoid biosynthesis (SG4; MYB4, MYB5). The identification of MYB4 and -5 was based on phylogenetic (**Figure 2**) and amino acid sequence analysis, including the presence of the EAR repression motif in the C-terminus (Supplementary Figure 2). The plant-specific EAR domain occurs in other TF families besides the R2R3-MYBs (Kagale and Rozwadowski, 2010), but alignment of confirmed and candidate AtMYB4-like R2R3-MYB-EAR repressors for flavonoid biosynthesis from a range of species showed the (P/L)DLNL(E/D)L version of the motif to be strongly conserved, and the sequence PDLNL(E/D)L is present in MYB4, MYB5 and characterized phenylpropanoid repressors from Arabidopsis, maize, and petunia. There are dominant white bulb phenotypes in onion and a color-inhibiting factor encoded by the *I* locus, which is homozygous dominant and conditions white bulbs (El-Shafie and Davis, 1967). Therefore, it would be interesting to investigate repressive regulatory factors in relation to this.

### Divergence of R2R3-MYB Anthocyanin Regulators within the Asparagales

Flavonoid regulatory genes have been previously characterized from only three monocot families, the Poaceae, Liliaceae, and Orchidaceae. The anthocyanin R2R3-MYBs activators from dicot species separate phylogenetically from those of the Poaceae and Orchidaceae (**Figure 2**; Supplementary Figure 5). The dicot sequences form the cluster termed SG6 (containing the anthocyanin regulator PAP1) based on the Arabidopsis R2R3-MYB gene family, while the Poaceae and Orchidaceae cluster with Arabidopsis SG5 (containing the PA regulator TT2). The absence of SG6 sequences from the Poaceae and Orchidaceae may suggest divergent evolution of the two MYB clades during the origins of dicots and monocots. However, the *MYB* genes from Asiatic lily (Liliaceae) and onion (Allioideae) cluster in the SG6/PAP1 clade. Thus, based on available sequence data it appears that the R2R3-MYBs that regulate anthocyanins have diverged within Asparagales evolution during formation of the Orchidaceae and Allioideae. Most phylogenetic analysis places the Orchidaceae as the sister group to the rest of the Asparagales, being one of the two initial phylogenetic branches (Angiosperm Phylogeny Group III, 2009), so divergence could well have occurred after this branching. The placement of the onion and Asiatic lily sequences with dicot rather than Orchidaceae/Poaceae sequences is not seen for the target genes of the flavonoid biosynthetic pathway. Rather, the biosynthetic gene sequences form phylogenetic trees close to the current suggested species taxonomic relationships (Supplementary Figure 6 shows a representative tree, for DFR). Additionally, the other flavonoid-related MYBs identified, for SG4 and SG7, group closely with the similar sequences from both dicots and the Poaceae (the only other monocot family for which sequences have been characterized).

SG5/C1-TT2 clade members were not identified in the approximately 250,000 onion Non-Redundant sequences. Nor have they been published for Asiatic lily, even though it may be expected that PA production is regulated by an R2R3-MYB in most monocots, as it is in the Poaceae (Himi and Noda, 2005; Himi et al., 2012) and dicot species (Nesi et al., 2001; Bogs et al., 2007; Hancock et al., 2012; Liu et al., 2014) studied to date. It may be that RNA sequencing for the Liliales and Asparagales species has not been conducted on samples from tissues rich in PAs. Interestingly, seed coats are typically rich in PAs but PAs have been functionally replaced by phytomelans in *Allium* and other taxa of Asparagales, but not in the Orchidaceae (Boesewinkel and Bouman, 1995; Fritsch and Friesen, 2002; Chase, 2004). Full genome sequences for species such as onion and Asiatic lily (∼130 and 900-fold larger, respectively, than that of Arabidopsis) are not yet available but sequencing of the gene rich regions is in progress for onion^1^. Additional DNA sequence resources for monocot species other than the Poaceae may also help to address the lack of reported PAP1-like sequences in some monocot species.

The basis for the absence of identified PAP1/SG6 clade members in the Poaceae and Orchidaceae is unknown. While additional DNA sequencing may identify members of this clade in such species, no PAP1-like R2R3-MYBs have been identified in the rice genome based on analysis of the phylogenies in Katiyar et al. (2012) and nor are any apparent in current transcriptome databases for various orchid species (the authors’ unpublished analysis). It seems unlikely that PAP1-like R2R3-MYBs arose independently in dicots, the Liliales and the Asparagales – i.e., convergent evolution after the separation from the Orchidaceae. More likely is that there has been neofunctionalization of the C1-TT2 (SG5) clade in the Poaceae and Orchidaceae with the subsequent loss of the PAP1 clade. The presence of regulators of both anthocyanins and PAs in the same clade in grasses is consistent with this theory. Furthermore in this scenario, both clades would have been retained in the Liliaceae, Allioideae, and dicots, with separation of function developing for anthocyanin and PA regulation. Alternatively, the SG6 clade could have evolved from the SG5 MYBs to regulate anthocyanins, whilst SG5 genes were retained to regulate PA synthesis, reflected by the two subclades of genes associated with anthocyanins and PAs within SG5 in grasses (**Figure 2**). To address this question fully, further characterisation of the anthocyanin and PA regulators is required from diverse monocot and gymnosperm species.

Finally, it is worth noting that some phylogenetic differences in the anthocyanin regulators were translated into differential activities in the transient assays. In contrast to MYB1, the maize MYB regulator Zm-C1 was not able to activate pigmentation in onion even when its bHLH partner was co-introduced (Supplementary Figure 4). Indeed, the maize anthocyanin regulators have been observed to inhibit pigmentation when bombarded into onion (personal communication, Dr. David Collings). Also, Zm-C1 (-/+ its bHLH partner) was not able to complement the snapdragon anthocyanin *MYB* mutant (the authors’ unpublished observation). Whether this reflects evolutionary divergence or different lineages for the regulators awaits further analysis. In the first instance, it would be interesting test the anthocyanin regulators from other lineages within the Asparagales.

## Conclusion

We have shown that MYB1 is a positive regulator of anthocyanin production in onion and we have demonstrated its utility as a molecular breeding tool. We have also identified a candidate MYB regulator for flavonols, the other key flavonoid metabolites in onion, as well as candidate MYB factors for repressive activities in the regulation of flavonoid or phenylpropanoid metabolism. These genes will be useful to identify classical loci in onion and will help in wider phylogenetic and regulatory studies in monocot lineages.

## Author Contributions

KS, HN, FK, CE, DB, KD, and NA conceived of and conducted experiments and/or analyzed and interpreted data; KS, DB, KD, and NA isolated, identified and/or analyzed gene sequences; JM, MP-J, and RC generated transcriptome resources; KD, KS, DB, and JM conceived of the project; KS, NA, and KD prepared the manuscript. All authors read and approved the manuscript.

## Conflict of Interest Statement

The authors declare that the research was conducted in the absence of any commercial or financial relationships that could be construed as a potential conflict of interest.

## Footnotes

1 http://www.oniongenome.net

## References

[B1] Albert N. W. Arathoon S. Collette V. E. Schwinn K. E. Jameson P. E. Lewis D. H. (2010). Activation of anthocyanin synthesis in *Cymbidium* orchids: variability between known regulators. *Plant Cell Tissue Organ Cult.* 100 355–360. 10.1007/s11240-009-9649-0

[B2] Albert N. W. Davies K. M. Lewis D. H. Zhang H. Montefiori M. Brendolise C. (2014a). A conserved network of transcriptional activators and repressors regulates anthocyanin pigmentation in eudicots. *Plant Cell* 26 962–980. 10.1105/tpc.113.122069 24642943 PMC4001404

[B3] Albert N. W. Davies K. M. Schwinn K. E. (2014b). Gene regulation networks generate diverse pigmentation patterns in plants. *Plant Signal. Behav.* 9:e29526 10.4161/psb.29526 PMC4205132 25763693

[B4] Albert N. W. Lewis D. H. Zhang H. Irving L. J. Jameson P. E. Davies K. M. (2009). Light-induced vegetative anthocyanin pigmentation in Petunia. *J. Exp. Bot.* 60 2191–2202. 10.1093/jxb/erp097 19380423 PMC2682507

[B5] Albert N. W. Lewis D. H. Zhang H. Schwinn K. E. Jameson P. E. Davies K. M. (2011). Members of an R2R3-MYB transcription factor family in Petunia are developmentally and environmentally regulated to control complex floral and vegetative pigmentation patterning. *Plant J.* 65 771–784. 10.1111/j.1365-313X.2010.04465.x 21235651

[B6] Allan A. C. Hellens R. P. Laing W. A. (2008). MYB transcription factors that colour our fruit. *Trends Plant Sci.* 13 99–102. 10.1016/j.tplants.2007.11.012 18280199

[B7] Angiosperm Phylogeny Group III (2009). An update of the Angiosperm Phylogeny Group classification for the orders and families of flowering plants: APG III. *Bot. J. Linn. Soc.* 161 105–121. 10.1016/j.jep.2015.05.035

[B8] Baldwin S. Revanna R. Thomson S. Pither-Joyce M. Wright K. Crowhurst R. (2012). A toolkit for bulk PCR-based marker design from next-generation sequence data: application for development of a framework linkage map in bulb onion (*Allium cepa* L.). *BMC Genomics* 13:637 10.1186/1471-2164-13-637 PMC3534495 23157543

[B9] Ballester A. R. Molthoff J. de Vos R. Hekkert B. Orzaez D. Fernandez-Moreno J. P. (2010). Biochemical and molecular analysis of pink tomatoes: deregulated expression of the gene encoding transcription factor SlMYB12 leads to pink tomato fruit color. *Plant Physiol.* 152 71–84. 10.1104/pp.109.147322 19906891 PMC2799347

[B10] Boesewinkel F. D. Bouman F. (1995). “The seed: structure and function,” in *Seed Development and Germination* eds Kigel J. Galili G. (New York, NY: Marcel Dekker Inc.) 13–14.

[B11] Bogs J. Jaffé F. W. Takos A. M. Walker A. R. Robinson S. P. (2007). The grapevine transcription factor VvMYBPA1 regulates proanthocyanidin synthesis during fruit development. *Plant Physiol.* 143 1347–1361. 10.1104/pp.106.093203 17208963 PMC1820911

[B12] Burr F. A. Burr B. Scheﬄer B. E. Blewitt M. Wienand U. Matz E. C. (1996). The maize repressor-like gene intensifier1 shares homology with the r1/b1 multigene family of transcription factors and exhibits missplicing. *Plant Cell* 8 1249–1259. 10.2307/3870299 8776895 PMC161237

[B13] Casati P. Walbot V. (2005). Differential accumulation of maysin and rhamnosylisoorientin in leaves of high-altitude landraces of maize after UV-B exposure. *Plant Cell Environ.* 28 788–799. 10.1111/j.1365-3040.2005.01329.x

[B14] Cavallini E. Matus J. T. Finezzo L. Zenoni S. Loyola R. Guzzo F. (2015). The phenylpropanoid pathway is controlled at different branches by a set of R2R3-MYB C2 repressors in grapevine. *Plant Physiol.* 167 1448–1470. 10.1104/pp.114.256172 25659381 PMC4378173

[B15] Chase M. W. (2004). Monocot relationships: an overview. *Am. J. Bot.* 91 1645–1655. 10.3732/ajb.91.10.1645 21652314

[B16] Chiou C.-Y. Yeh K.-W. (2008). Differential expression of MYB gene (OgMYB1) determines color patterning in floral tissue of oncidium gower ramsey. *Plant Mol. Biol.* 66 379–388. 10.1007/s11103-007-9275-3 18161007

[B17] Crowhurst R. N. Gleave A. P. MacRae E. A. Ampomah-Dwamena C. Atkinson R. G. Beuning L. L. (2008). Analysis of expressed sequence tags from Actinidia: applications of a cross species EST database for gene discovery in the areas of flavor, health, color and ripening. *BMC Genomics* 9:351 10.1186/1471-2164-9-351 PMC2515324 18655731

[B18] Czemmel S. Stracke R. Weisshaar B. Cordon N. Harris N. N. Walker A. R. (2009). The grapevine R2R3-MYB transcription factor VvMYBF1 regulates flavonol synthesis in developing grape berries. *Plant Physiol.* 151 1513–1530. 10.1104/pp.109.142059 19741049 PMC2773091

[B19] Davies K. M. Albert N. W. Schwinn K. E. (2012a). From landing lights to mimicry: the molecular regulation of flower colouration and mechanisms for pigmentation patterning. *Funct. Plant Biol.* 39 619–638. 10.1071/FP12195 32480814

[B20] Davies K. M. Deroles S. C. Boase M. R. Hunter D. A. Schwinn K. E. (2012b). “Biolistics-based gene silencing in plants,” in *Methods in Molecular Biology* eds Sudowe S. Reske-Kunz A. (Heidelberg: Springer-Verlag) 63–74. 10.1007/978-1-62703-110-3_6 23104334

[B21] Davies K. M. Espley R. V. (2013). Opportunities and challenges for the metabolic engineering of plant secondary metabolite pathways for improved human health characters in fruit and vegetable crops. *N. Z. J. Crop Hortic. Sci.* 41 154–177. 10.1080/01140671.2013.793730

[B22] Drummond A. J. Ashton B. Buxton S. Cheung M. Cooper A. Duran C. (2011). *Geneious v5.4*. Available at: http://www.geneious.com/

[B23] Duangjit J. Bohanec B. Chan A. P. Town C. D. Havey M. J. (2013). Transcriptome sequencing to produce SNP-based genetic maps of onion. *Theor. Appl. Genet.* 126 2093–2101. 10.1007/s00122-013-2121-x 23689743

[B24] Duangjit J. Welsh K. Wise M. L. Bohanec B. Havey M. J. (2014). Genetic analyses of anthocyanin concentrations and intensity of red bulb color among segregating haploid progenies of onion. *Mol. Breed.* 34 75–85. 10.1007/s11032-014-0018-2

[B25] Dubos C. Gourrierec J. L. Baudry A. Huep G. Lanet E. Debeaujon I. (2008). MYBL2 is a new regulator of flavonoid biosynthesis in *Arabidopsis thaliana*. *Plant J.* 55 940–953. 10.1111/j.1365-313X.2008.03564.x 18532978

[B26] Edgar R. C. (2004). MUSCLE: multiple sequence alignment with high accuracy and high throughput. *Nucleic Acids Res.* 32 1792–1797. 10.1093/nar/gkh340 15034147 PMC390337

[B27] El-Shafie M. Davis G. (1967). Inheritance of bulb color in the onion (*Allium cepa* L.). *Hilgardia* 38 607–622. 10.3733/hilg.v38n17p607

[B28] Feller A. MacHemer K. Braun E. L. Grotewold E. (2011). Evolutionary and comparative analysis of MYB and bHLH plant transcription factors. *Plant J.* 66 94–116. 10.1111/j.1365-313X.2010.04459.x 21443626

[B29] Ferreyra M. L. F. Casas M. I. Questa J. I. Herrera A. L. Deblasio S. Wang J. (2012). Evolution and expression of tandem duplicated maize flavonol synthase genes. *Front. Plant Sci.* 3:101 10.3389/fpls.2012.00101 PMC3360202 22654889

[B30] Ferreyra M. L. F. Rius S. Emiliani J. Pourcel L. Feller A. Morohashi K. (2010). Cloning and characterization of a UV-B-inducible maize flavonol synthase. *Plant J.* 62 77–91. 10.1111/j.1365-313X.2010.04133.x 20059741

[B31] Fritsch R. M. Friesen N. (2002). “Evolution, domestication and taxonomy,” in *Allium Crop Science: Recent Advances* eds Rabinowitch H. D. Currah L. (New York, NY: CAB International) 5–30.

[B32] Gavazzi G. Mereghetti M. Consonni G. Tonelli C. (1990). Sn a light-dependent and tissue-specific gene of Maize the genetic basis of its instability. *Genetics* 125 193–200. 2341031 10.1093/genetics/125.1.193 PMC1204002

[B33] Gleave A. P. (1992). A versatile binary vector system with a T-DNA organisational structure conducive to efficient integration of cloned DNA into the plant genome. *Plant Mol. Biol.* 20 1203–1207. 10.1007/BF00028910 1463857

[B34] Goff S. Cone K. Chandler V. L. (1992). Functional analysis of the transcriptional activator encoded by the maize B gene: evidence for a direct functional interaction between two classes of regulatory proteins. *Genes Dev.* 6 864–875. 10.1101/gad.6.5.864 1577278

[B35] Gould K. S. (2004). Nature’s Swiss army knife: the diverse protective roles of anthocyanins in leaves. *J. Biomed. Biotechnol.* 2004 314–320. 10.1155/S1110724304406147 15577195 PMC1082902

[B36] Gray J. Bevan M. Brutnell T. Buell C. R. Cone K. Hake S. (2009). A recommendation for naming transcription factor proteins in the grasses. *Plant Physiol.* 149 4–6. 10.1104/pp.108.128504 19126689 PMC2613739

[B37] Gray J. Caparros-Ruiz D. Grotewold E. (2012). Grass phenylpropanoids: regulate before using! *Plant Sci.* 184 112–120. 10.1016/j.plantsci.2011.12.008 22284715

[B38] Green F. N. Baur R. Thomson M. McCarthy L. (1997). An example of chartreuse skin colour in onion (*Allium cepa* L.) cultivar Greenella. *Genet. Resour. Crop Evol.* 44 491–493. 10.1023/A:1008649521256

[B39] Grotewold E. (2006). The genetics and biochemistry of floral pigments. *Annu. Rev. Plant Biol.* 57 761–780. 10.1146/annurev.arplant.57.032905.105248 16669781

[B40] Guindon S. Gascuel O. (2003). A simple, fast and accurate algorithm to estimate large phylogenies by maximum likelihood. *Syst. Biol.* 52 696–704. 10.1080/10635150390235520 14530136

[B41] Hancock K. R. Collette V. Fraser K. Greig M. Xue H. Richardson K. (2012). Expression of the R2R3 MYB transcription factor TaMYB14 from *Trifolium arvense* activates proanthocyanidin biosynthesis in the legumes *T. repens* and *Medicago sativa*. *Plant Physiol.* 159 1204–1220. 10.1104/pp.112.195420 22566493 PMC3387705

[B42] Hichri I. Barrieu F. Bogs J. Kappel C. Delrot S. Lauvergeat V. (2011). Recent advances in the transcriptional regulation of the flavonoid biosynthetic pathway. *J. Exp. Bot.* 62 2465–2483. 10.1093/jxb/erq442 21278228

[B43] Higo K. Ugawa Y. Iwamoto M. Korenaga T. (1999). Plant cis-acting regulatory DNA elements (PLACE) database: 1999. *Nucleic Acids Res.* 27 297–300. 10.1093/nar/27.1.297 9847208 PMC148163

[B44] Himi E. Noda K. (2005). Red grain colour gene (R) of wheat is a Myb-type transcription factor. *Euphytica* 143 239–242. 10.1007/s10681-005-7854-4

[B45] Himi E. Yamashita Y. Haruyama N. Yanagisawa T. Maekawa M. Taketa S. (2012). Ant28 gene for proanthocyanidin synthesis encoding the R2R3 MYB domain protein (Hvmyb10) highly affects grain dormancy in barley. *Euphytica* 188 141–151. 10.1007/s10681-011-0552-5

[B46] Kagale S. Rozwadowski K. (2010). Small yet effective. *Plant Signal. Behav.* 5 691–694. 10.4161/psb.5.6.11576 20505346 PMC3001561

[B47] Katiyar A. Smita S. Lenka S. K. Rajwanshi R. Chinnusamy V. Bansal K. C. (2012). Genome-wide classification and expression analysis of MYB transcription factor families in rice and *Arabidopsis*. *BMC Genomics* 13:544 10.1186/1471-2164-13-544 PMC3542171 23050870

[B48] Kenel F. Eady C. Brinch S. (2010). Efficient *Agrobacterium tumefaciens*-mediated transformation and regeneration of garlic (*Allium sativum*) immature leaf tissue. *Plant Cell Rep.* 29 223–230. 10.1007/s00299-009-0814-z 20099065

[B49] Khar A. Jakse J. Havey M. J. (2008). Segregations for onion bulb colors reveal that red is controlled by at least three loci. *J. Am. Soc. Hortic. Sci.* 133 42–47.

[B50] Kim S. Bang H. Yoo K. S. Pike L. M. (2006). Identification of the fourth allele of the ANS (anthocyanidin synthase) gene and its effect on red color intensity in onions (*Allium cepa*). *Euphytica* 149 45–51. 10.1007/s10681-005-9053-8

[B51] Kim S. Binzel M. L. Park S. H. Yoo K. S. Pike L. M. (2004a). Inactivation of DFR (dihydroflavonol 4-reductase) gene transcription results in blockage of anthocyanin production in yellow onions (*Allium cepa*). *Mol. Breed.* 14 253–263. 10.1023/B:MOLB.0000047770.92977.04

[B52] Kim S. Binzel M. L. Yoo K. S. Park S. Pike L. M. (2004b). Pink (P), a new locus responsible for a pink trait in onions (*Allium cepa*) resulting from natural mutations of anthocyanidin synthase. *Mol. Genet. Genomics* 272 18–27. 10.1007/s00438-004-1041-5 15480791

[B53] Kim S. Jones R. Yoo K. S. Pike L. M. (2004c). Gold color in onions (*Allium cepa*): a natural mutation of the chalcone isomerase gene resulting in a premature stop codon. *Mol. Gen. Genom.* 272 411–419. 10.1007/s00438-004-1076-7 15503141

[B54] Kim S. Jones R. Yoo K. S. Pike L. M. (2005a). The L locus, one of complementary genes required for anthocyanin production in onions (*Allium cepa*), encodes anthocyanidin synthase. *Theor. Appl. Genet.* 111 120–127. 10.1007/s00122-005-2000-1 15856159

[B55] Kim S. Yoo K.-S. Pike L. M. (2005b). The basic color factor, the C locus, encodes a regulatory gene controlling transcription of chalcone synthase genes in onions (*Allium cepa*). *Euphytica* 142 273–282. 10.1007/s10681-005-2239-2

[B56] Kranz H. D. Denekamp M. Greco R. Jin H. Leyva A. Meissner R. C. (1998). Towards functional characterisation of the members of the R2R3-MYB gene family from *Arabidopsis thaliana*. *Plant J.* 16 263–276. 10.1046/j.1365-313x.1998.00278.x 9839469

[B57] Kuhl J. C. Cheung F. Yuan Q. Martin W. Zewdie Y. McCallum J. (2004). A unique set of 11,008 onion expressed sequence tags reveals expressed sequence and genomic differences between the monocot orders Asparagales and Poales. *Plant Cell* 16 114–125. 10.1105/tpc.017202 14671025 PMC301399

[B58] Leitch I. Kahandawala I. Suda J. Hanson L. Ingrouille M. J. Chase M. W. (2009). Genome size diversity in orchids: consequences and evolution. *Ann. Bot.* 104 469–481. 10.1093/aob/mcp003 19168860 PMC2720655

[B59] Liu C. Jun J. Dixon R. A. (2014). MYB5 and MYB14 play pivotal roles in seed coat polymer biosynthesis in *Medicago truncatula*. *Plant Physiol.* 165 1424–1439. 10.1104/pp.114.241877 24948832 PMC4119029

[B60] Ludwig S. R. Habera L. F. Dellaporta S. L. Wessler S. R. (1989). Lc a member of the maize R gene family responsible for tissue-specific anthocyanin production encodes a protein similar to transcriptional activators and contains the Myc-homology region. *Proc. Natl. Acad. Sci. U.S.A.* 86 7092–7096. 10.1073/pnas.86.18.7092 2674946 PMC298000

[B61] Luo J. Butelli E. Hill L. Parr A. Niggeweg R. Bailey P. (2008). AtMYB12 regulates caffeoyl quinic acid and flavonol synthesis in tomato: expression in fruit results in very high levels of both types of polyphenol. *Plant J.* 56 316–326. 10.1111/j.1365-313X.2008.03597.x 18643978

[B62] Ma H. Pooler M. Griesbach R. (2008). Ratio of Myc and Myb transcription factors regulates anthocyanin production in orchid flowers. *J. Am. Soc. Hortic. Sci.* 133 133–138.

[B63] Ma H. Pooler M. Griesbach R. (2009). Anthocyanin regulatory/structural gene expression in Phalaenopsis. *J. Am. Soc. Hortic. Sci.* 134 88–96.

[B64] Martin C. Butelli E. Petroni K. Tonelli C. (2011). How can research on plants contribute to promoting human health? *Plant Cell* 23 1685–1699. 10.1105/tpc.111.083279 21586682 PMC3123949

[B65] Masuzaki S. Shigyo M. Yamauchi N. (2006). Complete assignment of structural genes involved in flavonoid biosynthesis influencing bulb color to individual chromosomes of the shallot (*Allium cepa* L.). *Genes Genet. Syst.* 81 255–263. 10.1266/ggs.81.255 17038797

[B66] Mehrtens F. Kranz H. Bednarek P. Weisshaar B. (2005). The *Arabidopsis* transcription factor MYB12 is a flavonol-specific regulator of phenylpropanoid biosynthesis. *Plant Physiol.* 138 1083–1096. 10.1104/pp.104.058032 15923334 PMC1150422

[B67] Morohashi K. Casas M. I. Falcone-Ferreyra M. L. Mejia-Guerra M. K. Pourcel L. Yilmaz A. (2012). A genome-wide regulatory framework identifies maize pericarp color1 controlled genes. *Plant Cell* 24 2745–2764. 10.1105/tpc.112.098004 22822204 PMC3426112

[B68] Nakatsuka A. Yamagishi M. Nakano M. Tasaki K. Kobayashi N. (2009). Light-induced expression of basic helix-loop-helix genes involved in anthocyanin biosynthesis in flowers and leaves of Asiatic hybrid lily. *Sci. Hortic.* 121 84–91. 10.1093/pcp/pcq011

[B69] Nesi N. Jond C. Debeaujon I. Caboche M. Lepiniec L. (2001). The *Arabidopsis* TT2 gene encodes an R2R3 MYB domain protein that acts as a key determinant for proanthocyanidin accumulation in developing seed. *Plant Cell* 13 2099–2114. 10.2307/3871430 11549766 PMC139454

[B70] Patil B. S. Pike L. M. Yoo K. S. (1995). Variation in the quercetin content in different colored onions (*Allium cepa* L.). *J. Am. Soc. Hortic. Sci.* 120 909–913.

[B71] Ramsay N. A. Glover B. J. (2005). MYB-bHLH-WD40 protein complex and the evolution of cellular diversity. *Trends Plant Sci.* 10 63–70. 10.1016/j.tplants.2004.12.011 15708343

[B72] Schwinn K. Venail J. Shang Y. Mackay S. Alm V. Butelli E. (2006). A small family of MYB-regulatory genes controls floral pigmentation intensity and patterning in the genus Antirrhinum. *Plant Cell* 18 831–851. 10.1105/tpc.105.039255 16531495 PMC1425845

[B73] Shang Y. Schwinn K. E. Bennett M. J. Hunter D. A. Waugh T. L. Pathirana N. N. (2007). Methods for transient assay of gene function in floral tissues. *Plant Methods* 3 1 10.1186/1746-4811-3-1 PMC1781449 17207290

[B74] Shelton D. Stranne M. Mikkelsen L. Pakseresht N. Welham T. Hiraka H. (2012). Transcription factors of Lotus: regulation of isoflavonoid biosynthesis requires coordinated changes in transcription factor activity. *Plant Physiol.* 159 531–547. 10.1104/pp.112.194753 22529285 PMC3375922

[B75] Stracke R. Favory J.-J. Gruber H. Bartelniewoehner L. Bartels S. Binkert M. (2010). The *Arabidopsis* bZIP transcription factor HY5 regulates expression of the PFG1/MYB12 gene in response to light and ultraviolet-B radiation. *Plant Cell Environ.* 33 88–103. 10.1111/j.1365-3040.2009.02061.x 19895401

[B76] Stracke R. Ishihara H. Huep G. Barsch A. Mehrtens F. Niehaus K. (2007). Differential regulation of closely related R2R3-MYB transcription factors controls flavonol accumulation in different parts of the *Arabidopsis thaliana* seedling. *Plant J.* 50 660–677. 10.1111/j.1365-313X.2007.03078.x 17419845 PMC1976380

[B77] Stracke R. Werber M. Weisshaar B. (2001). The R2R3-MYB gene family in *Arabidopsis thaliana*. *Curr. Opin. Plant Biol.* 4 447–456. 10.1016/S1369-5266(00)00199-0 11597504

[B78] Tonelli C. Consonni G. Dolfini S. F. Dellaporta S. L. Viotti A. Gavazzi G. (1991). Genetic and molecular analysis of Sn, a light-inducible, tissue specific regulatory gene in maize. *Mol. Gen. Genet.* 225 401–410. 10.1007/BF00261680 1673220

[B79] Tonelli C. Dolfini S. Ronchi A. Consonni G. Gavazzi G. (1994). Light inducibility and tissue specificity of the R gene family in maize. *Genetica* 94 225–234. 10.1007/BF01443436

[B80] Yamagishi M. Shimoyamada Y. Nakatsuka T. Masuda K. (2010). Two R2R3-MYB genes, homologs of petunia AN2, regulate anthocyanin biosyntheses in flower tepals, tepal spots and leaves of Asiatic hybrid lily. *Plant Cell Physiol.* 51 463–474. 10.1093/pcp/pcq011 20118109

[B81] Zimmermann I. M. Heim M. A. Weisshaar B. Uhrig J. F. (2004). Comprehensive identification of *Arabidopsis thaliana* MYB transcription factors interacting with R/B-like BHLH proteins. *Plant J.* 40 22–34. 10.1111/j.1365-313X.2004.02183.x 15361138

