# Supplementary material for: The Onion (Allium cepa L.) R2R3-MYB Gene MYB1 Regulates Anthocyanin Biosynthesis
Source: Front Plant Sci. 2016 Dec 9;7:1865. doi: 10.3389/fpls.2016.01865 (PMC5146992; doi:10.3389/fpls.2016.01865)
Supplement: Supplementary file 1 [file Data_Sheet_1.PDF]

Supplementary Material

The onion (*Allium cepa* L.) *R2R3-MYB* gene MYB1 regulates anthocyanin biosynthesis

Kathy E. Schwinn, Hanh Ngo, Fernand Kenel, David A. Brummell, Nick W. Albert, John A. McCallum, Meeghan Pither-Joyce, Ross N. Crowhurst, Colin Eady, and Kevin M. Davies*

***Corresponding Author: kevin.davies@plantandfood.co.nz**

1 Supplementary Table 1. Primers used in the study (5′ to 3′).

**General primers**	
AAP	GGC CAC GCG TCG ACT AGT ACG GGI IGG GII GGG IIG
AUAP	GGC CAC GCG TCG ACT AGT AC
cDNA Synthesis	GAC TCG AGT CGA CAT CGA T_{17}
SP	GAC TCG AGT CGA CAT CGA
Myb1 3'-RACE	
K454	AAY GAR ATM AAR AAY TAY TGG AA
K456	GGI AAY AGR TGG TCI CTN ATW GC
Myb1 5'-RACE	
K474 cDNA Synthesis	AAT CTA GCA TGC ATA AGC AC
K475	TCT GGT TGC ACC ATT ACT GC
K476	TCT TCA TCC TCG GTT GTT TC
K477	TAC ACG GTG ATG ATG AAT GC
Myb1 Genome walking	
MYB1-I	GCT CCA TGC TCC TTT TCG TAC ACC ATC AC
MYB1-O	GTC CCG CAC GAG ATG GAA CGG AAC TC
Myb1 cDNA and genomic clone isolation (to span full coding sequence); overexpression vector pKES22	
K480	CAA TCC TTC TTC ACG TTG TAT AC
K474	AAT CTA GCA TGC ATA AGC AC
Myb1 RNAi knockdown vector pHMN3 (underlined sequences are incorporated restriction enzyme sites)	
K594	ATC GAT CTA GAG GTG CAA CCA GAA GAG TC
K515	ATC GCA GAT CTG GAG ATT ATT ATG AGA TG
**Primers for qPCR**	
MYB1 K550	ACT GCG ATG GCC AAT GAA ACA AAT
MYB1 K551	GCC TGG TCC GTC ATA TCA AAC TCA
CHS K653	ACC AGG TGG AGG CGA AGG TG
CHS K654	ACA ACA CGC ATG CGC TCG ACA
DFR K554	ACC GTT CGC GCC ACT CTC AG
DFR K555	TTG GGG TAG CGA CAT GGA AAA CAG
Ubiquitin K599	TCT GAT TGT GCT TGT GGT GGT T
Ubiquitin K600	AAA GCG GAA ATT AAG ATG ATG TTG C
GAPDH K597	CCA TGT TTG TTG TTG GTG TGA ATG AG
GAPDH K598	TGG TGC AGC TAG CGT TGG AG AC
***HptII* primers for transgenic garlic analysis**	
OPR_139 hptII-f1	CCG CAA GGA ATC GGT CAA TA
OPR_140 hptII-r1	CCC AAG CTG CAT CAT CGA AA
**Overexpression vector pNASA3 (cDNA from ABPL cultivar)**	
NA96	CCA TCG ATA TGG CGC TCT CAG CTT CC
NA97	CGG GAT CCT CAT CGC TTC CCT ATA GCT TTA C

2 Supplementary Figures

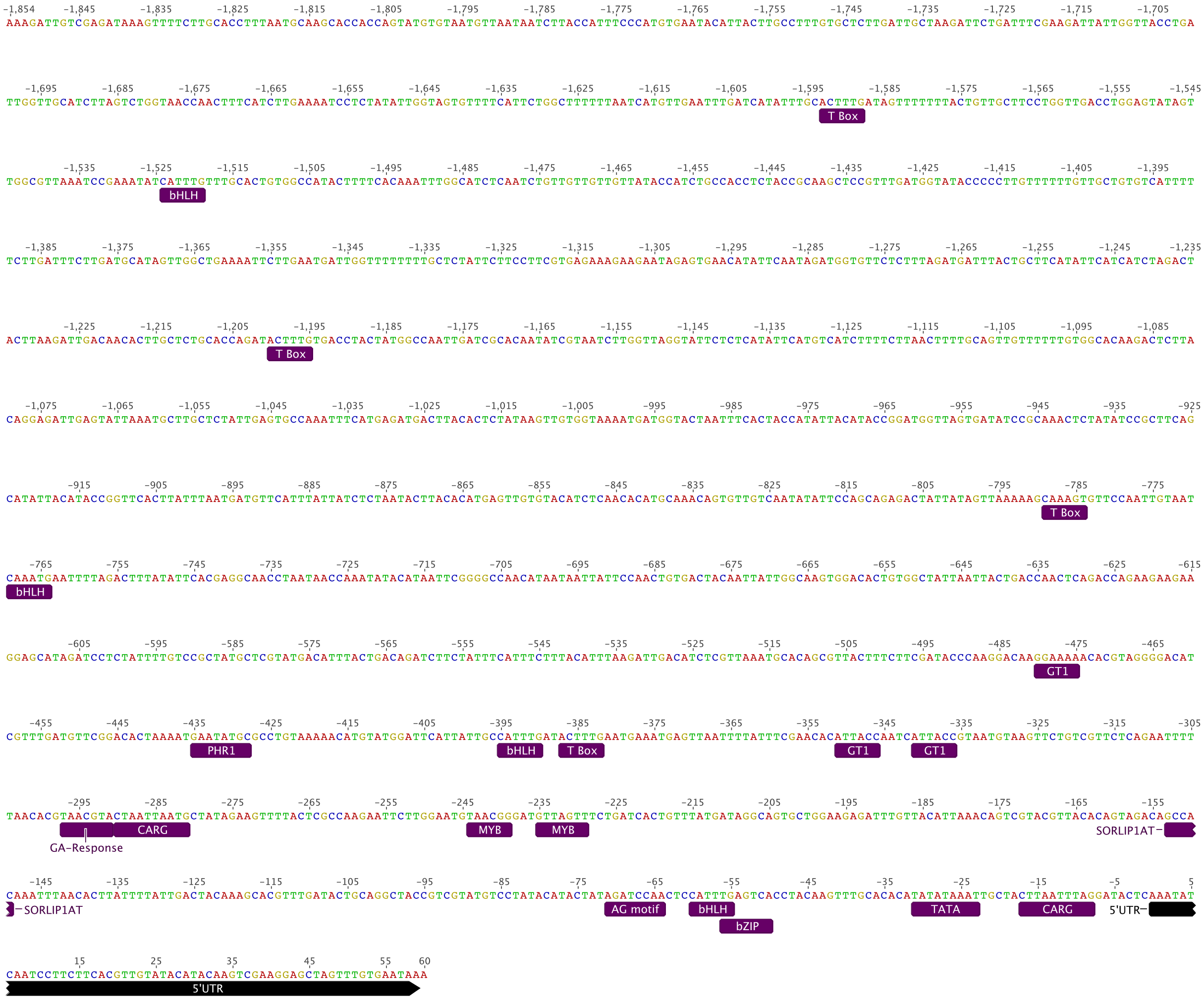

**AG-motif (AGATCCAA):** GATA-type ZnFn recognition motif; Present in genes induced by various stresses
**bHLH/Myc consensus (CANNTG):** "R response element" for bHLH recognition
**bZIP consensus (TGAGTCA):** Motif for bZIP recognition
**CARG Box (CWWWWWWWWG):** Motif for MADS box binding
**GT1 CONSENSUS (GRWAAW)**: Consensus GT-1 binding site in many light-regulated genes; GT-1 can stabilize the TATA box complex
**GARE2OSREP1 (TAACGTA):** Gibberellin-responsive element
**MYB1LEPR (GTTAGTT) and MYBCOREATCYCB1 (AACGG):** Myb recognition motif
**PHR-1 binding motif (GNATATNC):** PHR1 transcription factor recognition motif
**WBOXATNPR1 (TTGAC):** "W-box" recognized by salicylic acid (SA)-induced WRKY proteins
**SORLIP1AT (GCCAC):** Sequence most over-represented in light-induced promoters in *Arabidopsis*
**TBOXATGAPB (ACTTTG):** "T-box" related to light-activated gene transcription

Supplementary Figure 1. The Sequence and potential *cis* elements of the genomic region upstream from the known cDNA sequence for *MYB1*. Notable sequence motifs for transcriptional regulation were identified using the PLACE website analysis. Details of these are given in the accompanying table.

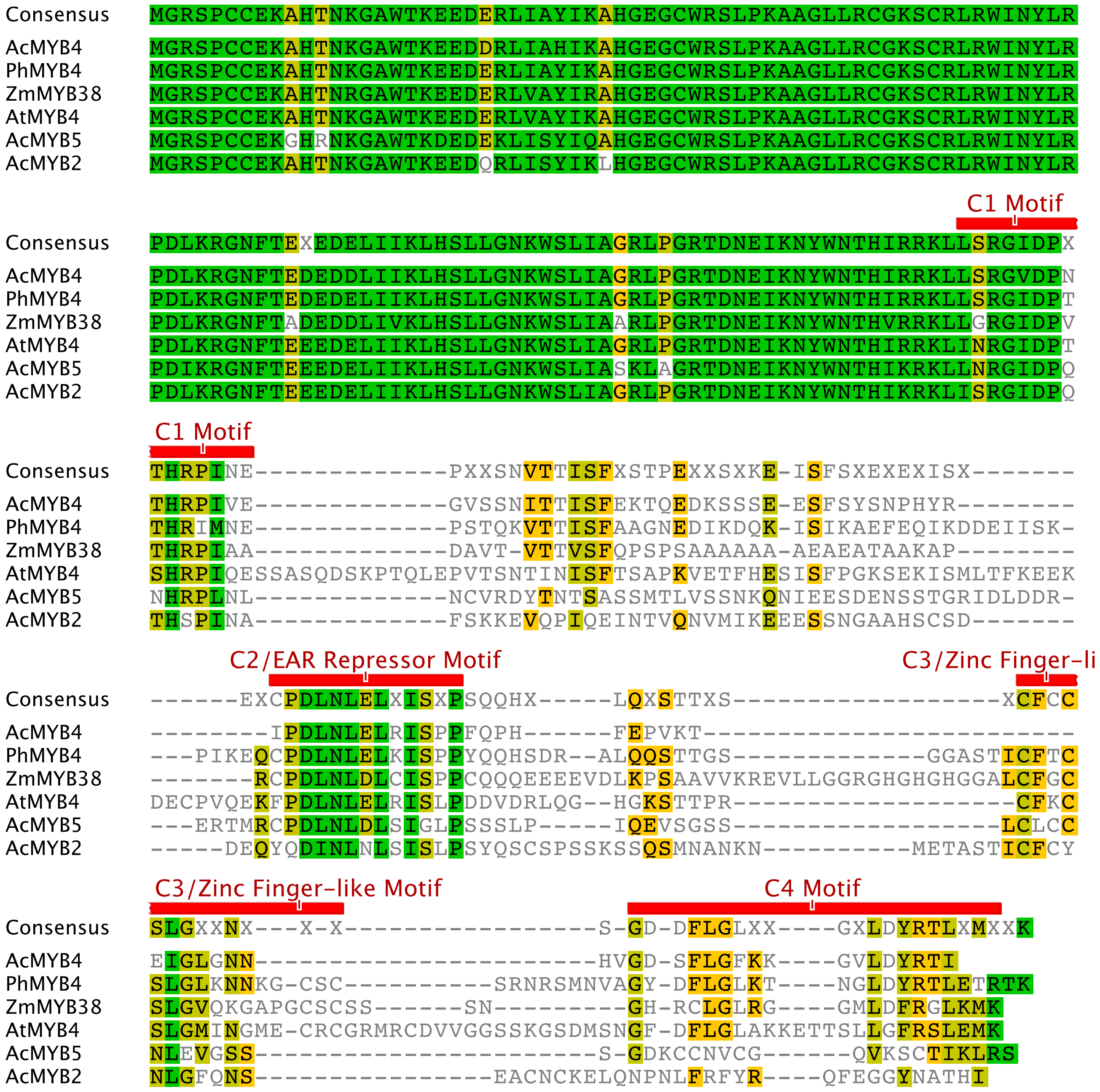

Supplementary Figure 2. Onion (*Allium cepa*) R2R3MYB sequences with motifs conserved in characterised repressor R2R3MYBs of petunia (PhMYB4), maize (ZmMYB38), Arabidopsis (AtMYB4). ClustalW was used for the alignment and the conserved repressor motifs identified by Cavallini *et al.* (2015, Plant Physiology 167: 1448) are indicated on the consensus.

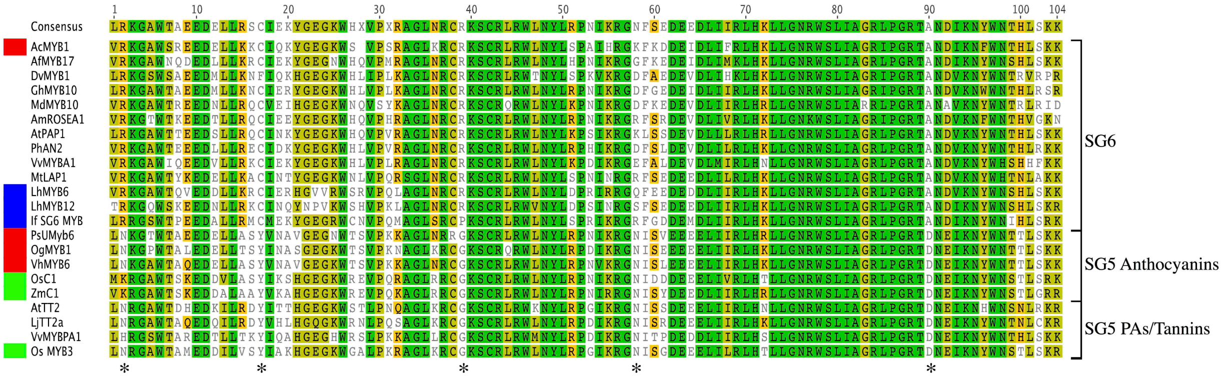

Supplementary Figure 3. ClustalW alignment of 22 selected R2R3-MYB sequences from SG5 and SG6, showing only the conserved R2R3 domain. A numbered consensus sequence is shown at the top. Amino acid positions showing consistent differences among the clades are indicated with asterisks. Asparagales, Poaceae and other moncot species are flagged with red, green and blue, respectively. The domain for interaction with bHLH partners is from positions 65-84.

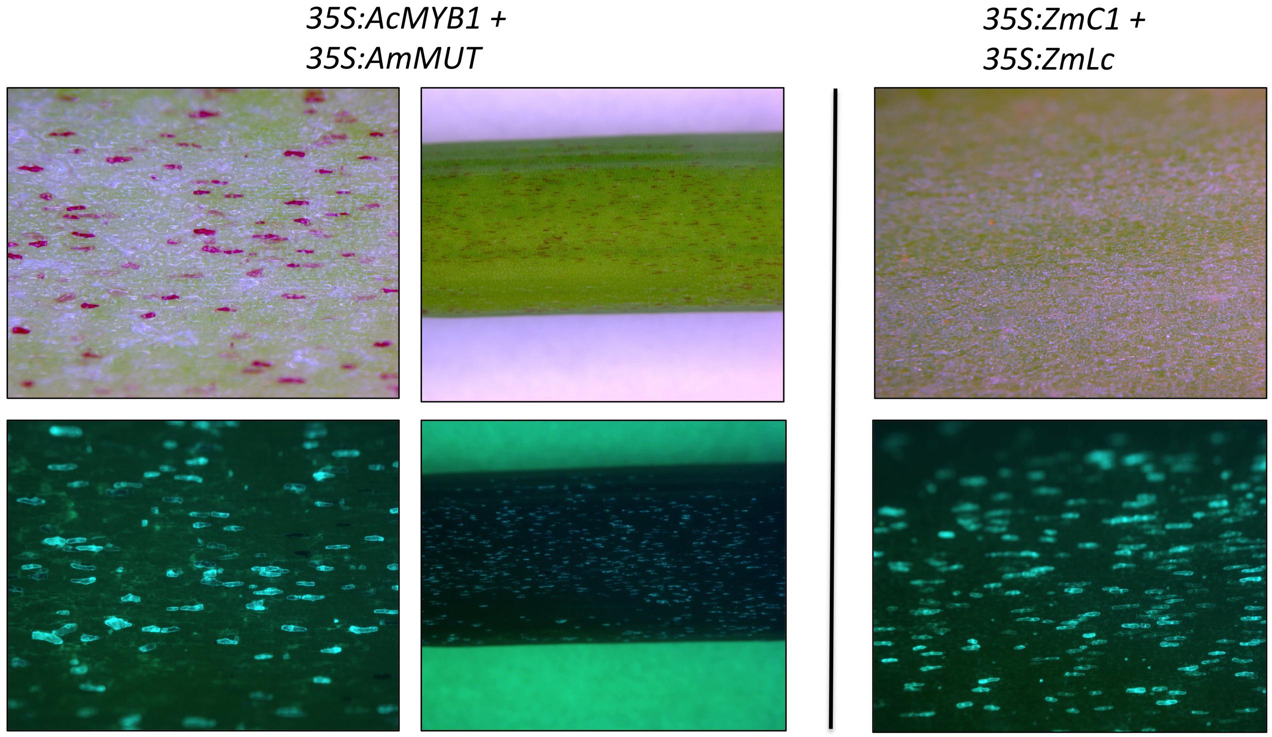

Supplementary Figure 4. Overexpression of anthocyanin regulators in onion (*Allium cepa*). A GFP construct was included in both experiments as an internal transformation control. Left panels show seedling tissue bombarded with constructs for onion MYB1 and a bHLH anthocyanin regulator from *Antirrhinum majus*, MUTABILIS. Seedlings are at the stage before bulbing occurs, and the middle panel shows a view across the whole seedling width. Red cells have colocalised GFP fluorescence. Right panels show seedling tissue bombarded with constructs for maize MYB (C1) and bHLH (Lc) anthocyanin regulators. GFP fluorescence shows transformation was successful, but no red cells were observed. Top panels are images viewed in white light and bottom panels in blue light for GFP fluorescence.

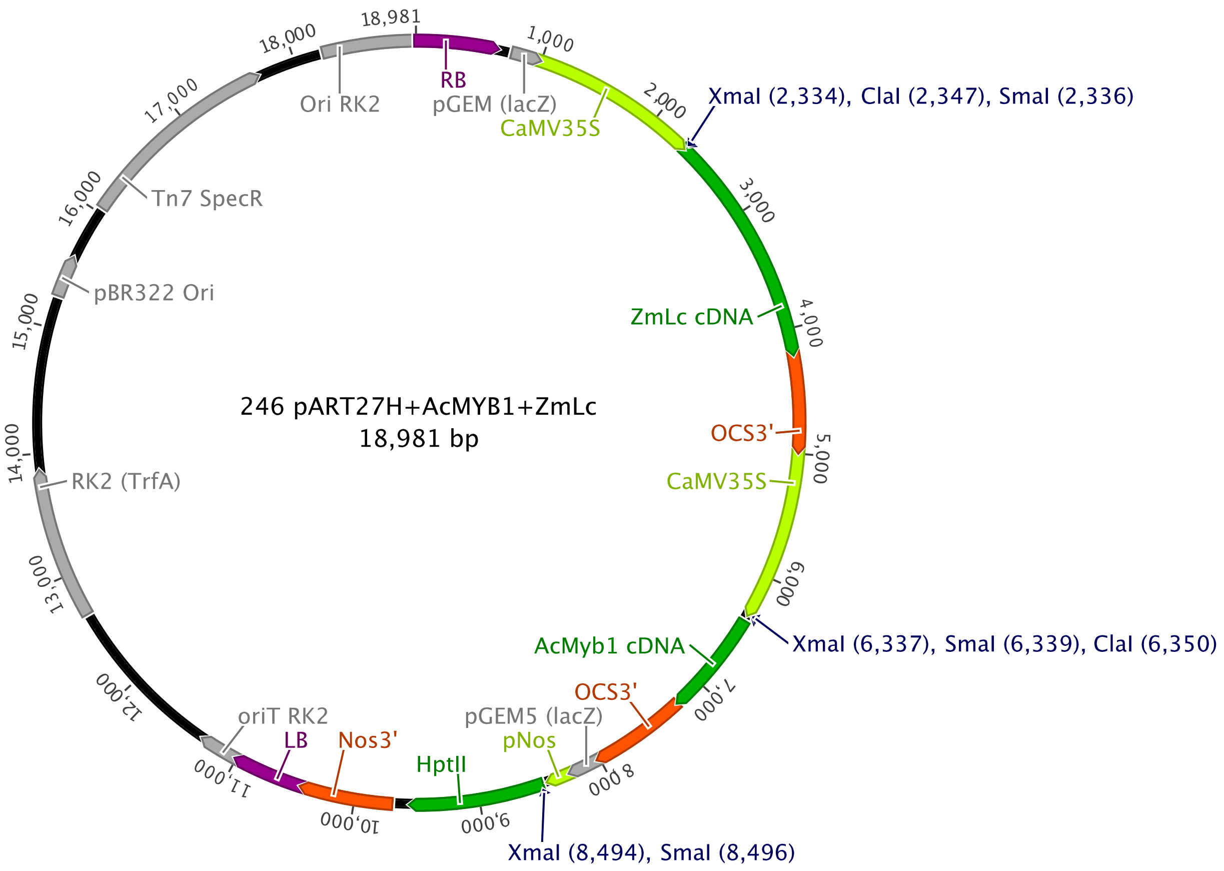

Supplementary Figure 5. Vector used for production of stably transformed garlic (*Allium sativum*) plants, containing *35S:AcMYB1*, *35S:ZmLc* and *NOS:HPTII* in the pART27 backbone vector.

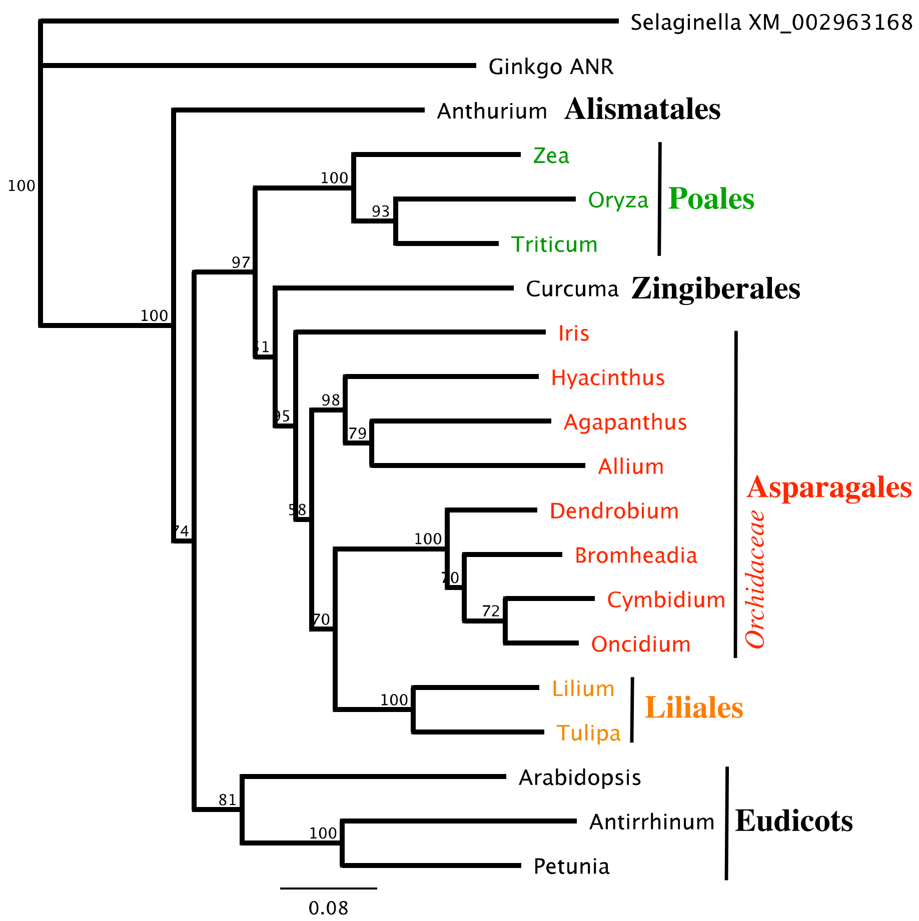

Supplementary Figure 6. Phylogenetic relationship of dihydroflavonol 4-reductase (DFR) sequences from a range of species. A phylogenetic tree was formed using the full-length deduced DFR amino acid sequences from a range of monocot species and asterid and rosid eudicots, and related sequences from *Ginkgo* and the spikemoss *Selaginella* to root the tree. Analysis used the Geneious R6 (Biomatters Ltd, Auckland, New Zealand) suite of software, with the alignment created using ClustalW and phylogenies using the Jukes-Cantor/Neighbor-Joining method of Geneious Tree Builder (Drummond *et al.* 2011; Edgar 2004). The scale bar represents number of substitutions per site, and the numbers next to the nodes are bootstrap values from 1000 replicates. The plant orders or major groups are also provided.

GenBank database accession numbers are *Agapanthus praecox* (BAE78769); *Allium cepa DFR-A* (AAO63026); *Anthurium andraeanum* (ABC94578); *Antirrhinum majus* (P14721); *Arabidopsis thaliana* (BAA85261); *Bromheadia finlaysoniana* (AAB62873); *Curcuma alismatifolia* (ADK62520); *Cymbidium hybrida* (AAC17843); *Dendrobium* hybrid (AEF58500); *Ginkgo biloba* ANR (AAU95082); *Hyacinthus orientalis* (AFP58815); *Iris* x *hollandica* (BAF93896); *Lilium* (hybrid division I) (AAQ83576); *Oncidium* Gower Ramsey (AAY32602); *Oryza sativa* (BAA36182); *Petunia* x *hybrida* (AF233639); *Selaginella moellendorffii* DFR-like (XM_002963168); *Triticum aestivum* (AAV83987); *Tulipa gesneriana* (BAH98155); *Zea mays* (NP_001152467).
